# Compressive Stress-Assisted Drilling for Delamination Suppression in C/SiC Composites: Mechanism and Experimental Validation

**DOI:** 10.3390/ma19153230

**Published:** 2026-07-29

**Authors:** Qiudong Zhang, Zhenyu Shi, Cunwen Wang, Guodong Shao, Xianzhi Zhang

**Affiliations:** 1 School of Mechanical Engineering, Hebei University of Technology, Tianjin 300130, China; 13258856433@163.com; 2 Key Laboratory of High Efficiency and Clean Mechanical Manufacture (Ministry of Education), School of Mechanical Engineering, Shandong University, Jinan 250061, China; shaoguodong@163.com; 3 Shandong Lianxing Energy Group Company, Jining 272500, China; wagncunwen@163.com; 4 School of Computing and Engineering, The University of Huddersfield, Huddersfield HD1 3DH, UK; x.zhang@kingston.ac.uk

**Keywords:** C/SiC ceramic matrix composites (CMCs), hole-exit delamination, compressive stress-assisted drilling, strain energy release rate, graphite plate

## Abstract

Carbon-fiber-reinforced silicon carbide (C/SiC) ceramic matrix composites (CMCs) exhibit substantial application potential in the field of advanced industrial manufacturing, attributed to their inherent superiorities such as high specific strength, excellent high-temperature resistance, and prominent corrosion resistance. Nevertheless, hole-exit delamination is a critical defect in C/SiC composite drilling, which impairs the structural integrity and service reliability of components, restricting their engineering implementation. To address this issue, this study proposes and systematically investigates a compressive stress-assisted drilling method for delamination suppression via applying external compressive stress. Using the delamination factor for quantitative evaluation, comparative experiments were conducted under unassisted drilling, graphite-plate-assisted drilling without preload, and graphite-plate-assisted drilling with varying preload torques. The results indicate that compressive stress significantly mitigates delamination, with a maximum delamination factor reduction rate of 18.29%. Mechanistically, the compressive stress effectively controls delamination by suppressing Mode I crack propagation at the crack tip and elevating the critical strain energy release rate. Furthermore, this work elucidates that the essential role of the graphite plate is to provide a controllable in-plane pre-compressive stress field for the workpiece drilling zone.

## 1. Introduction

Carbon-fiber-reinforced silicon carbide (C/SiC) composites possess excellent high-temperature, corrosion and wear resistance [1,2,3], making them indispensable for aero-engine hot-end components and nuclear structural parts [4,5]. As summarized in the 2019 review by Diaz et al. [6], the number of annual publications on cutting and drilling of ceramic matrix composites nearly tripled in the five years before the publication of this review. However, high hardness and anisotropy lead to poor machinability. Drilling axial force easily triggers hole-exit delamination, which impairs component accuracy and reliability and restricts industrial application [7]. This study focuses on this key engineering problem.

To relieve hole-exit delamination during C/SiC drilling, two typical strategies have been proposed to suppress delamination. The first includes conventional milling, grinding [8,9,10], drilling [11,12,13] and non-traditional energy-field machining such as rotary ultrasonic machining [14,15,16,17,18], EDM [19], laser ablation [20,21,22] and abrasive waterjet cutting [23,24,25,26]. Clijsters et al. [27] optimized EDM parameters for conductive modified C/SiC to alleviate fiber pull-out damage; Jiao et al. [28] and Zhang et al. [29] implemented nanosecond laser blind-hole machining and quantified the influence of scanning speed on hole quality; Feng et al. [14] and Ding et al. [30] verified that rotary ultrasonic drilling effectively reduces axial cutting force and fiber tearing. The second low-cost mitigation strategy is graphite-plate-assisted drilling. Capello et al. [31] investigated the backing protection performance on CFRP laminates under fixed spindle speed and feed rate, proving that rigid graphite backing can reduce hole-exit delamination by 12–15%. This beneficial effect originates from the high compressive stiffness and uniform contact behavior of graphite plates, which avoids local indentation and stress concentration at the hole exit during tool penetration. For 2.5D woven C/SiC composites with the same interlock preform structure as the present specimen, Xing et al. [11] carried out backing contrast experiments and microscopic damage characterization. Their measurements confirmed that graphite constraint restrains out-of-plane bending of the bottom ply, delivering an 18% reduction in the maximum delamination factor. A series of classical critical thrust force models were established and continuously improved by Hocheng, Tsai and Tsao [32,33,34,35], whose theoretical systems take linear elastic fracture mechanics and thin-plate small-deflection bending theory as the dual theoretical basis. In their complete analytical frameworks, multiple key characteristic parameters are fully coupled, including workpiece laminate thickness, drill bit geometric size, composite elastic modulus, Poisson’s ratio and intrinsic Mode I critical strain energy release rate *G_IC_*. The theoretical derivation quantitatively correlates the passive contact support force provided by graphite backing plates with the critical axial thrust threshold that triggers Mode I interlaminar crack initiation. The unified model prediction indicates that when laminate geometric size and inherent fracture toughness *G_IC_* remain constant, increasing the elastic stiffness of the backing plate will continuously elevate the safe critical cutting load, thus delaying the onset of exit delamination defects.

Nevertheless, both approaches have clear deficiencies. Non-traditional machining is not suitable for mass production. Traditional graphite plates only produce fixed backing force from drill extrusion, so the delamination suppression effect cannot be quantitatively optimized. Meanwhile, existing theories offer inconsistent explanations of the plate’s working principle: Tsao [35] attributed the delamination suppression effect to the elevation of critical axial thrust for crack initiation, while Hocheng and Tsai explained the protection mechanism based on thin-plate bending restraint and strain energy release rate criteria. In contrast, Xing et al. [11] emphasized that the core function of the graphite plate lies in restraining bottom-layer push-out deformation and homogenizing edge tensile stress. To date, few unified stress-field-based mechanical interpretations have been proposed to reconcile these conflicting viewpoints, and the quantitative correlation between backing constraint stress, structural geometric parameters, and delamination evolution remains unclear.

The above limitations create an obvious research gap. Few studies introduce adjustable preload to generate variable in-plane compressive stress, and the quantitative relationship between preload and delamination degree has not been systematically investigated. The essential function of the graphite plate from the stress field perspective also remains unclear. To fill this gap, this work puts forward a testable hypothesis: adjustable preload can generate planar compressive stress that inhibits Mode I crack propagation, and the graphite plate merely acts as a load homogenization medium rather than the core factor suppressing delamination. This hypothesized mechanical explanation has not been verified in previous literature and requires combined theoretical derivation and comparative drilling tests for validation.

To verify the above hypothesis, this work proposes a compressive stress-assisted drilling method with adjustable fixture preload. The core objectives are to establish a crack evolution model under compressive stress, conduct comparative tests covering unassisted drilling, zero-preload graphite plate drilling and gradient preload-assisted drilling, and evaluate whether the proposed stress-based crack suppression mechanism holds true against experimental delamination data. The main innovation of this work lies in the design of a tunable preloading scheme that enables quantitative adjustment of workpiece internal stress; whether the corresponding compressive stress crack inhibition hypothesis is valid will be judged by the subsequent theoretical and experimental results, rather than treated as a confirmed fact in advance.

## 2. Theoretical Analysis of Drilling-Induced Delamination Defects

Carbon-fiber-reinforced silicon carbide (C/SiC) ceramic matrix composites (CMCs) are intrinsically anisotropic and heterogeneous materials. In the course of machining, the induced thermal stress exhibits a highly complex state, which arises from the elevation of cutting temperature and the dynamic fluctuations of machining environmental conditions—encompassing coolant properties, machine tool vibration, tool wear evolution, ambient temperature, and humidity levels. Collectively, these multifactorial variables exert a non-negligible influence on the initiation and propagation of delamination defects at the hole exit during machining, rendering it a formidable challenge to develop a comprehensive mathematical model that incorporates all contributing factors for the accurate characterization of the underlying delamination mechanism. Systematic analysis of delamination defects is performed from the viewpoint of energy conversion in this study, and a mathematical model describing delamination behaviors at the hole exit is developed and validated preliminarily.

### 2.1. Analysis of Forces Relevant to Delamination

During the drilling process of carbon fiber-reinforced silicon carbide (C/SiC) ceramic matrix composites, the workpiece domain to be machined is primarily subjected to two types of forces: one is the cutting force exerted by the brazed diamond abrasive drill bit, and the other is the interlaminar bonding force of the composite laminates. The drilling force can be decomposed into two components, namely, the axial drilling force and the radial force. Based on experimental findings and relevant literature reports, the magnitude of the radial force component is extremely small, and its influence on the initiation and propagation of delamination defects at the hole exit is negligible; therefore, subsequent mechanical behavior analysis focuses solely on the action effect of the axial drilling force.

**Figure 1:**
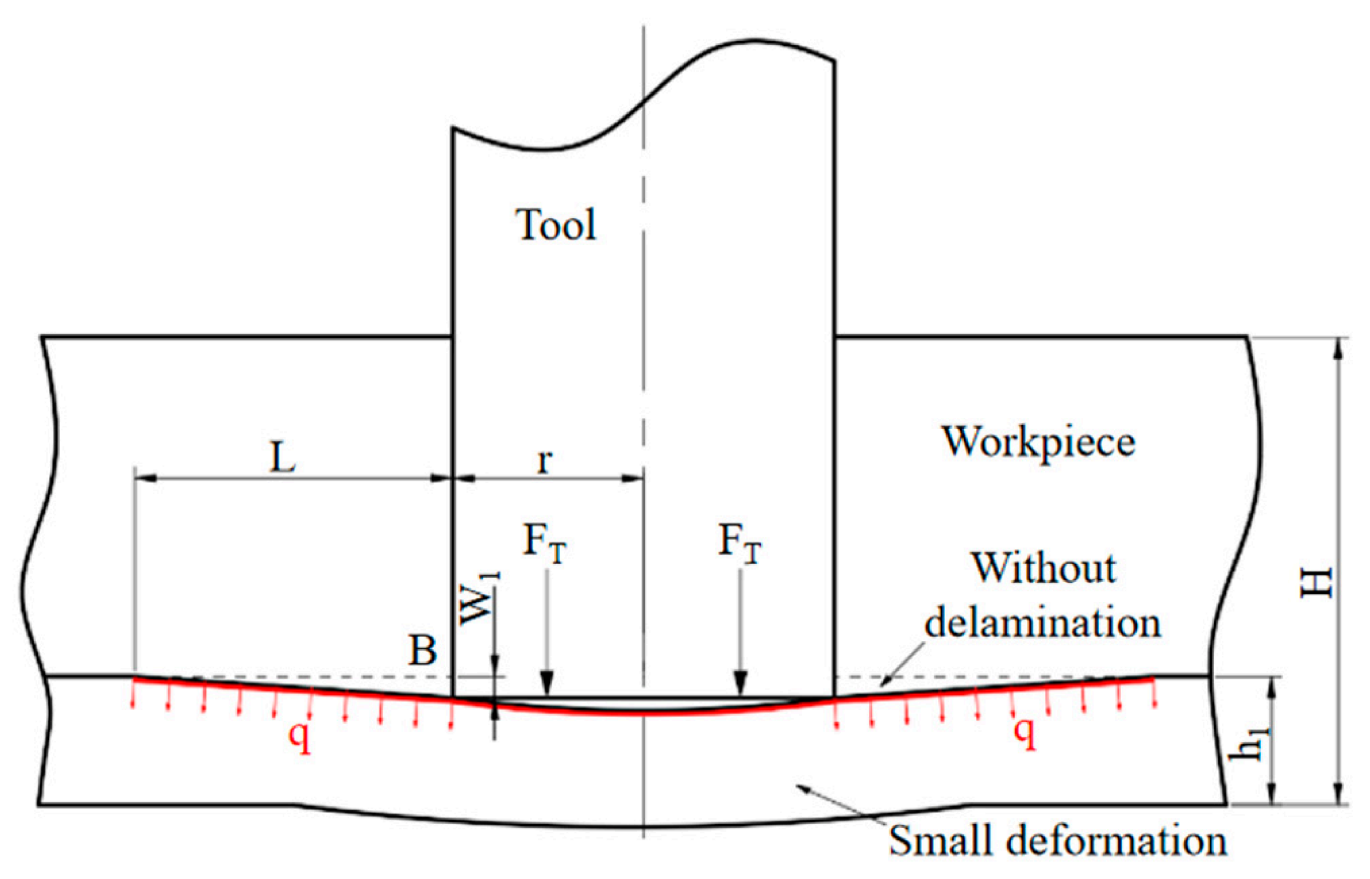
Small deformation.

**Figure 2:**
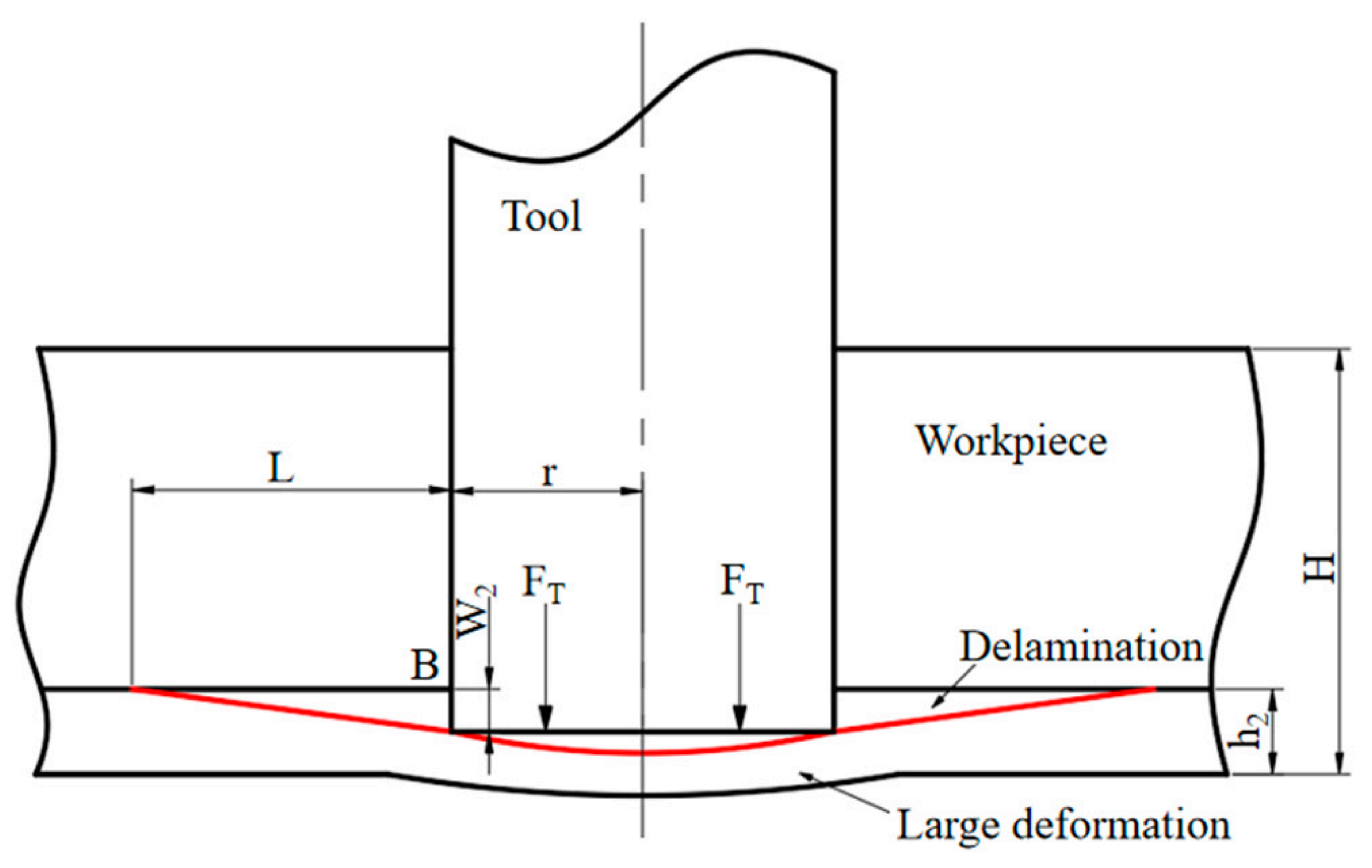
Large deformation.

As shown in Figure 1 and Figure 2, during the drilling process of carbon-fiber-reinforced silicon carbide (C/SiC) ceramic matrix composites, the uncut layer maintains a relatively large initial thickness *h*_1_ at the preliminary machining stage, which endows it with substantial deformation resistance. Correspondingly, only marginal elastoplastic deformation occurs in this region, and the interfacial bonding strength between the uncut layer and the adjacent upper ply is sufficient to preserve structural integrity, thereby effectively inhibiting the initiation of delamination damage. With the advancement of drilling operations, the thickness of the uncut layer gradually diminishes from *h*_1_ to *h*_2_, resulting in a significant reduction in structural stiffness and a notable attenuation of resistance to the axial drilling force. Consequently, the deformation magnitude of the uncut layer increases substantially. Once the induced deformation exceeds the critical threshold, the interlaminar bonding strength is no longer capable of withstanding the deformation-induced internal stress. This triggers the detachment of the uncut layer from the upper ply, thereby initiating the nucleation and propagation of interlaminar cracks and ultimately culminating in the formation of delamination defects at the hole exit.

In accordance with the Griffith Criterion in Mode I Fracture Mechanics, combined with the literature and experimental data reported by Zhang, G. D [36,37], the axial drilling thrust induces bending deformation (denoted by the red line in the figure) within the uncut layer, albeit the magnitude of such deformation is negligible in the initial machining stage. With the continuous advancement of the drilling process, the residual thickness of the uncut layer gradually decreases, which directly gives rise to a significant attenuation of its flexural rigidity and thereby a substantial reduction in the bending deformation resistance of the uncut layer. Under this premise, the bending deflection of the uncut layer increases progressively. This deformation evolution process is inherently correlated with the attenuation law of the load-bearing capacity of the uncut layer, laying the mechanical foundation for the subsequent initiation and propagation of delamination defects at the hole exit.

Based on the aforementioned analytical results, the formation mechanism of delamination defects at the hole exit can be delineated into the following sequential stages: First, under the action of the axial drilling force, the uncut layer undergoes initial elastic–plastic deformation; subsequently, with the continuous progression of the drilling process, the magnitude of deformation within the uncut layer increases progressively; finally, the interlaminar bonding strength of the carbon-fiber-reinforced silicon carbide (C/SiC) ceramic matrix composite is insufficient to withstand the deformation-induced internal stress, which triggers the propagation and mutual penetration of interlaminar cracks, thereby culminating in the formation of delamination defects at the hole exit.

### 2.2. Theoretical Modeling of Delamination Defects

#### 2.2.1. Establishment of Axial Drilling Force Analytical Model

**Figure 3:**
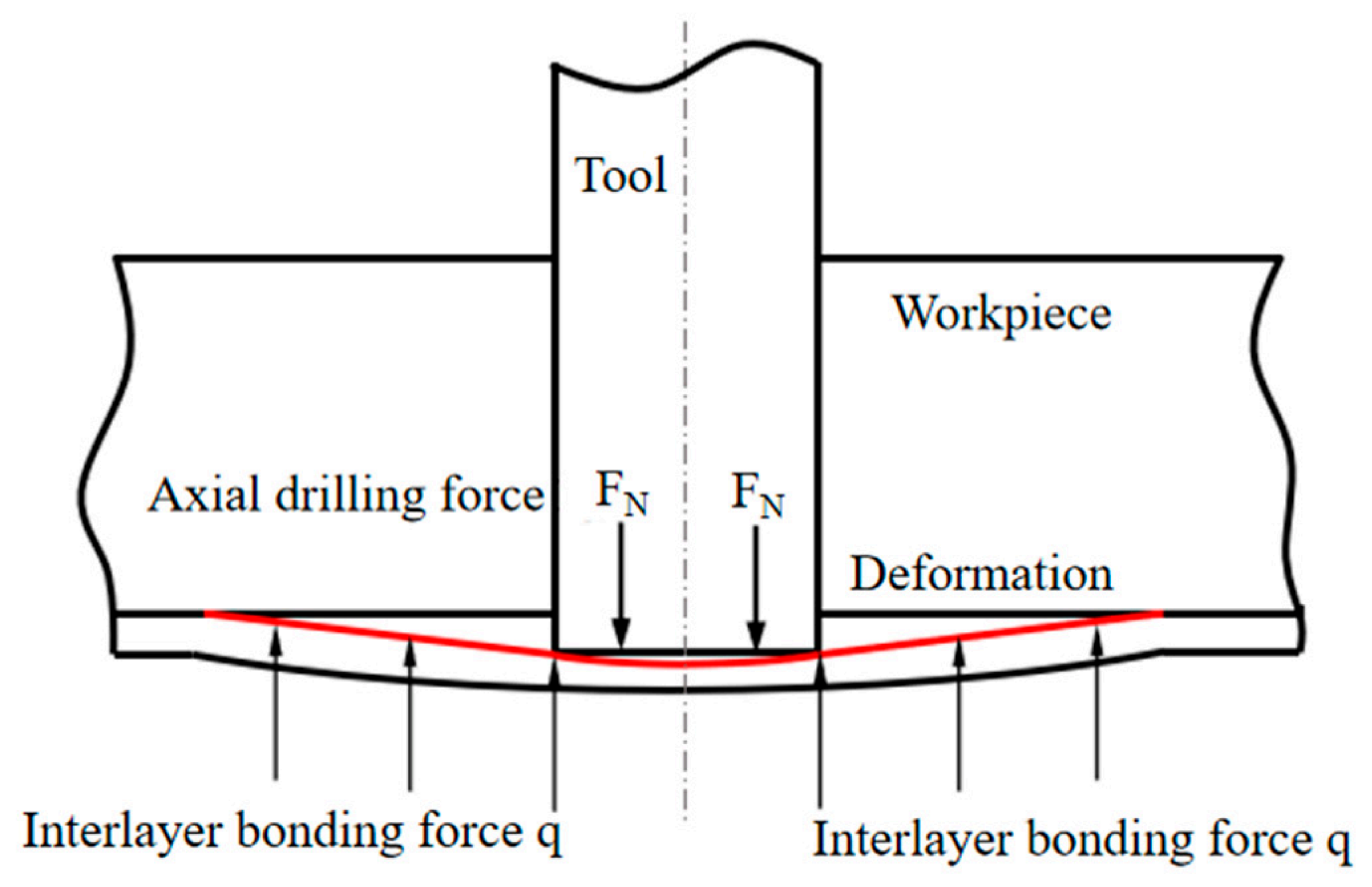
Schematic diagram of force analysis of work piece.

To intuitively characterize the correlation between uncut layer deformation and axial drilling force, the deformation behavior was amplified and schematically illustrated in Figure 3. In the present study, a layer-wise analytical model for hole-exit delamination defects was established. Specifically, the axial drilling force *F_N_* was simplified as a concentrated force, whereas the interlaminar bonding force *q* was idealized as a uniformly distributed load. The key drilling process parameters and their corresponding symbols involved in the theoretical modeling are summarized in Table 1.

Notably, the bending deformation of the workpiece domain to be machined necessitates the interlaminar bonding force between adjacent laminates to maintain a state of mechanical equilibrium. In accordance with the Griffith fracture criterion, Mode I cracks will be initiated when the downward tensile stress induced by deformation exceeds the interlaminar bonding strength of the composite. With the continuous propagation and interconnection of these cracks, delamination defects are ultimately formed at the hole exit.

It is widely acknowledged that axial drilling force dominates the initiation of exit delamination during composite drilling. When axial thrust exceeds the critical threshold, interlaminar cracks nucleate and eventually evolve into hole-exit delamination failure. Therefore, developing an analytical axial force model carries vital theoretical and engineering value, which can provide quantitative guidance for process optimization and delamination suppression of composite materials. In this work, an axial drilling force analytical model is established based on cutting-grinding mechanics and Usui’s grinding theory, combined with the geometric and material removal characteristics of brazed diamond abrasive drills.

**Figure 4:**
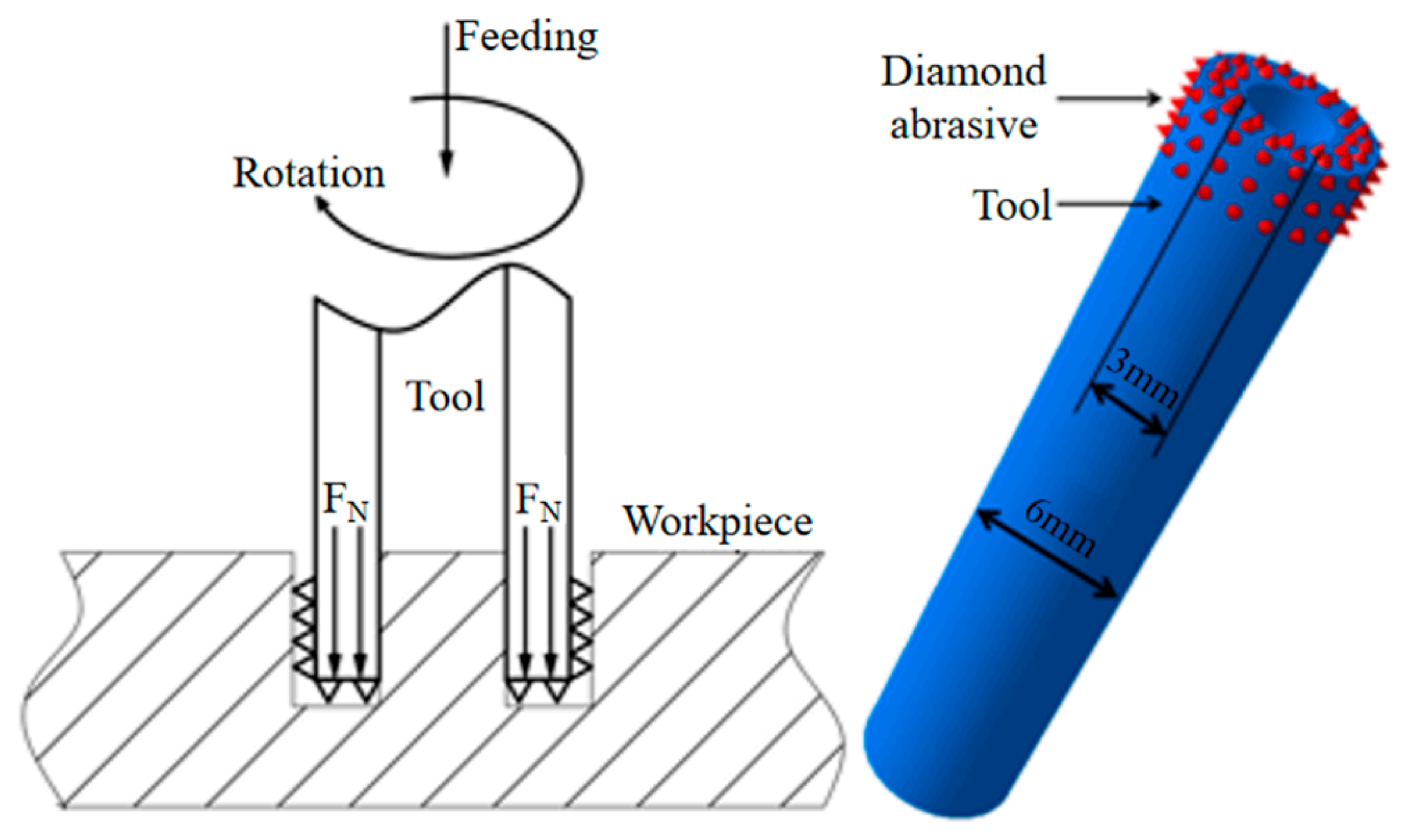
Model of hole-exit delamination.

As illustrated in Figure 4, the material removal behavior of brazed diamond drills drilling 2.5D woven C/SiC ceramic matrix composites is analogous to surface grinding. Hence, the grinding mechanical framework proposed by Eiji Usui [38] is adopted as the theoretical foundation to derive the cutting force formula.

**Figure 5:**
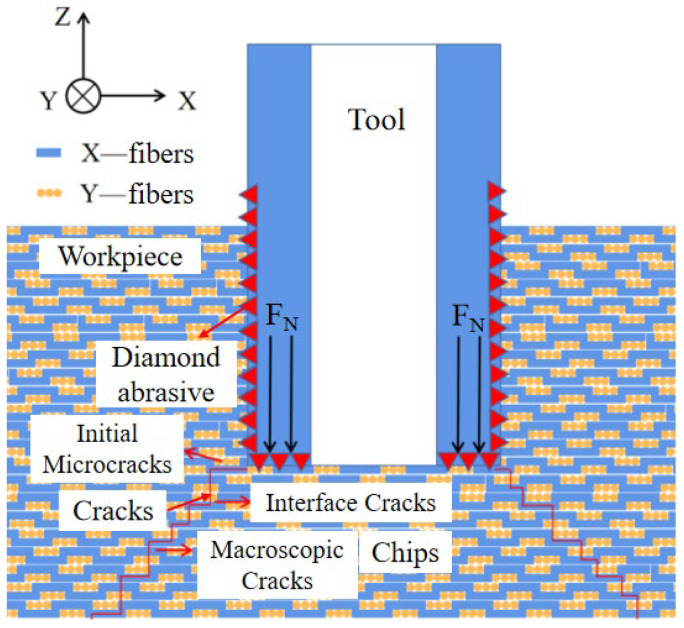
Schematic diagram of machining process.

In the force modeling shown in Figure 5, two geometric assumptions are adopted for the brazed diamond abrasive grains: each single abrasive grain has a fixed cone half-angle *γ*, and abrasives are uniformly distributed over the drill bit surface.

**Figure 6:**
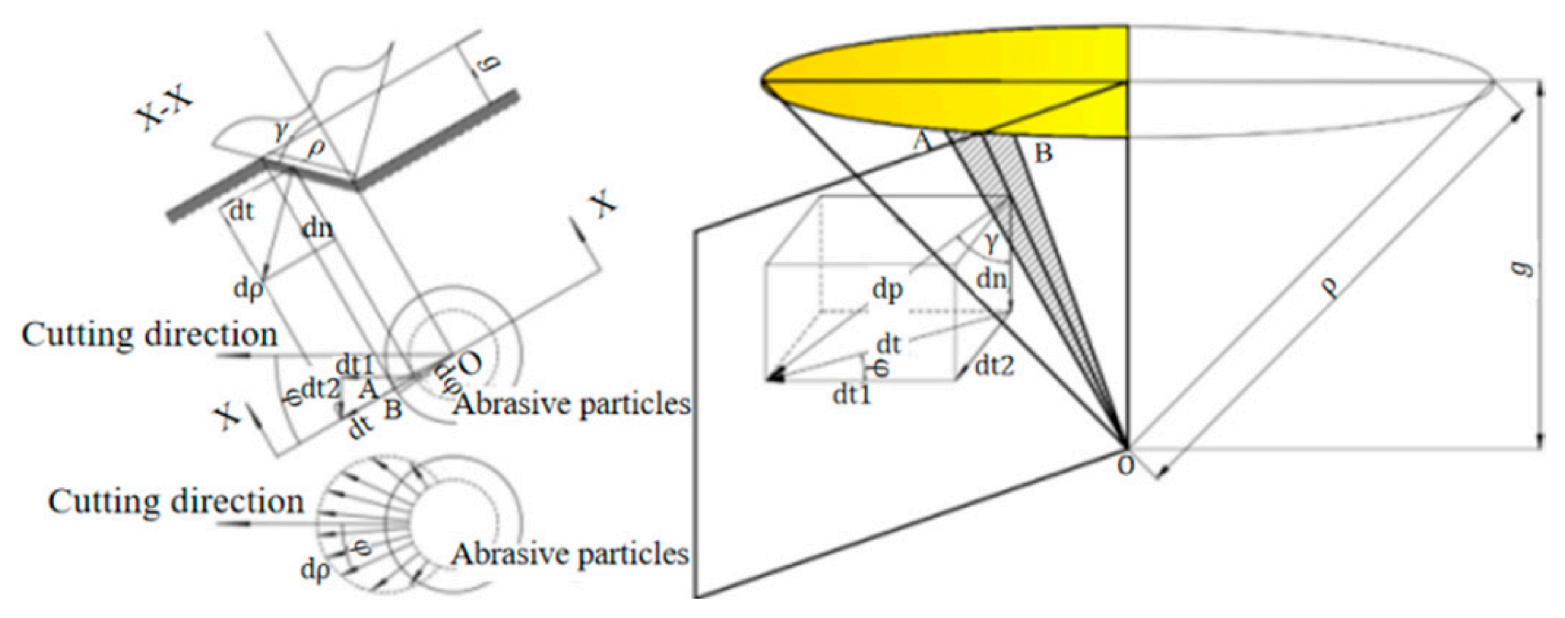
Stress analysis of abrasive particles.

Let *ρ* be the generatrix length of the conical cutting zone engaged with the workpiece. The infinitesimal micro-element area OAB shown in Figure 6 can be expressed as [39]:

(1) $$dA=\frac{1}{2}\rho^{2}\sin\gamma d_{\varphi }$$

where *dA* denotes differential area of microelement OAB; *ρ* denotes effective generatrix length participating in cutting; *γ* denotes abrasive cone half-angle; *φ* denotes circumferential angle between the microelement and cutting direction. The total axial force is derived by integrating the infinitesimal force *dp* acting on each microelement OAB (see Figure 6).

Two types of normal stress act on abrasive microelements, with opposite mechanical effects:

(1) *σ*: tensile/extrusion normal stress induced by drill cutting, which drives crack propagation (positive tensile stress);
(2) *σ*_0_: uniform external pre-compressive stress loaded by the fixture, which counteracts the cutting tensile stress and produces crack-tip closure (negative compressive stress).

The term *σ*_total_ = *σ* + *σ*_0_ is a unified algebraic superposition formula, where *σ*_0_ carries a negative sign in actual calculation to reflect compressive action. Increasing the magnitude of applied pre-compression enlarges the absolute value of negative *σ*_0_, reduces the net tensile equivalent stress *σ*_total_, and thereby lowers the resulting axial drilling force. This algebraic definition unifies the mathematical derivation and physical suppression mechanism, eliminating apparent contradiction between formula form and experimental trends.

The superimposed equivalent normal stress on micro-area *dA* is written as $\sigma_{\mathrm{total}}$ = *σ* + *σ*_0_. The infinitesimal cutting force borne by the microelement is:

(2) $$d_{p}=\sigma_{\mathrm{total}}\cos\gamma \cos\varphi \mathrm{dA}=\frac{1}{2}(\sigma +\sigma_{0})\rho^{2}\sin\gamma \cos\gamma \cos\varphi d_{\varphi }$$

where *σ* denotes tensile contact stress generated by drilling cutting; *σ*_0_ denotes external pre-compressive stress applied on the workpiece, the core adjustable control parameter of this work.

Orthogonal decomposition of Equation (2) yields the vertical component of infinitesimal force:

(3) $$d_{n}=d_{p}\sin\gamma =\frac{1}{2}(\sigma +\sigma_{0})\rho^{2}\sin^{2}\gamma \cos\gamma \cos\varphi d\varphi$$

Integrating over the full circumferential range [−π/2, π/2] gives the vertical downward force generated by one single active abrasive grain:

(4) $$F_{n}=\int_{\frac{-\pi }{2}}^{\frac{\pi }{2}}d_{n}=\int_{\frac{-\pi }{2}}^{\frac{\pi }{2}}\frac{1}{2}(\sigma +\sigma_{0})\rho^{2}\sin^{2}\gamma \cos\gamma \cos\varphi d\varphi =(\sigma +\sigma_{0})\rho^{2}\sin^{2}\gamma \cos\gamma$$

where *F_n_* denotes vertical load contributed by one cutting abrasive grain.

Let *j* denote the total number of abrasives simultaneously engaged in cutting, the resultant total axial drilling force reads:

(5) $$F_{N}=j\times F_{n}=j(\sigma +\sigma_{0})\rho^{2}\sin^{2}\gamma \cos\gamma$$

In actual drilling processes, the maximum feed per revolution is limited by the exposed height of diamond abrasives, which are constrained by the geometric and kinematic features of brazed diamond drills. An excessively large feed will cause ineffective cutting or abrasive breakage. Under this constraint, the geometric relation among feed rate, spindle speed and effective cutting generatrix length is:

(6) $$\rho \cos\gamma =\frac{f}{v}$$

where *f* denotes feed rate, and *v* denotes spindle rotation speed.

Substitute Equation (6) into Equation (5) to eliminate *ρ*:

(7) $$F_{N}=j(\sigma +\sigma_{0}){\left(\frac{f}{v}\right)}^{2}\sin\gamma \tan\gamma$$

Mathematically, Equation (7) presents a linear correlation between *F_N_* and the algebraic sum *σ* + *σ*_0_. Physically, *σ*_0_ is compressive stress with negative algebraic value. When the magnitude of applied pre-compression rises, $\left|\sigma_{0}\right|$ increases, the net tensile value of *σ* + *σ*_0_ decreases, and the predicted axial drilling force *F_N_* reduces accordingly. This theoretical deduction is fully consistent with the later experimental observation that elevated pre-compressive stress suppresses axial drilling load.

During drilling, as the drill approaches the hole exit, the bottom laminate bears axial load and tends to separate from the base material along the hole edge. This delamination initiation occurs at the critical state where axial drilling force balances interlaminar bonding strength. At this critical position, axial load induces downward displacement *X* of the loaded ply and triggers elastic deformation in the drilling-affected zone.

The mechanical work done by axial drilling force is converted into elastic strain energy stored in the deformed material and Mode I crack propagation energy. Based on energy conservation law, the energy balance relation at critical delamination onset is established as:

(8) $$G_{I}\mathrm{dA}=F_{N}\mathrm{dX}-\mathrm{dU}$$

where *G_I_* denotes Mode I strain energy release rate per unit crack area; *dA* denotes incremental crack expansion area; *F_N_* denotes axial drilling force; *dX* denotes infinitesimal downward displacement of the loaded laminate.

The incremental crack area is approximated as:

(9) $$\mathrm{dA}=\pi {\left(a+\mathrm{dA}\right)}^{2}$$

where *a* denotes initial crack radius; *da* denotes infinitesimal crack propagation increment.

The elastic strain energy stored in the circular deformed region beneath the drill is expressed as [34]:

(10) $$U=\frac{8\pi MX^{2}}{a^{2}}$$

where *M* denotes unit bending stiffness of composite laminate, calculated via thin-plate bending theory:

(11) $$M=\frac{Eh^{3}}{12(1-v^{2})}$$

where *E* denotes elastic modulus, *ν* denotes Poisson’s ratio and *h* denotes single ply thickness.

The downward ply displacement induced by axial drilling force is derived as [40]:

(12) $$X=\frac{F_{N}a^{2}}{16\pi M}$$

where *X* denotes vertical displacement of the bottom ply under axial thrust.

#### 2.2.2. Correlation Between Strain Energy Release Rate and Delamination Criteria

Built on the energy conversion model derived in Section 2.2.1, which quantifies the relation between axial drilling force and Mode I crack growth energy, this subsection further establishes the quantitative linkage among strain energy release rate *G_I_*, adjustable external pre-compressive stress *σ*_0_, and delamination initiation criterion. The explicit inclusion of controllable *σ*_0_ in the revised theoretical formulas strengthens the logical consistency between theoretical derivation and subsequent experimental characterization, and clarifies the physical mechanism of pre-compression assisted delamination suppression. The core goal is to form a quantitative judgment standard for hole-exit delamination nucleation and expansion, providing theoretical support for optimizing delamination suppression strategies by regulating *σ*_0_.

Substitute displacement Equation (12) into energy balance Equation (8) and rearrange to solve for *G_I_*:

(13) $$G_{I}=\frac{3{F_{N}}^{2}(1-v^{2})}{8\pi^{2}Eh^{3}}$$

Further substitute the axial force Formula (7) containing core variable *σ*_0_ into Equation (13):

(14) $$G_{I}=\frac{3{\left(j(\sigma +\sigma_{0}){\left(\frac{f}{v}\right)}^{2}\sin\gamma \tan\gamma \right)}^{2}(1-v^{2})}{8\pi^{2}Eh^{3}}$$

Mathematically, Equation (14) shows *G_I_* is positively correlated with the square of *σ* + *σ*_0_. From physical stress superposition, *σ*_0_ is negative compressive stress offsetting positive cutting tensile stress σ. Increasing the magnitude of applied pre-compression reduces the net tensile value of *σ* + *σ*_0_, which decreases both axial force *F_N_* and Mode I strain energy release rate *G_I_*. This interpretation reconciles the mathematical formulation with the physical suppression mechanism observed in tests.

Equation (14) directly incorporates the external pre-compressive stress *σ*_0_, the key research parameter of this work. This quantitative relation bridges machining mechanics (axial load regulated by *σ*_0_) and linear elastic fracture mechanics (Mode I crack driving energy *G_I_*), enabling quantitative evaluation of how adjusting pre-compression alters *G_I_*, critical fracture threshold *G_IC_*, and delamination initiation risk.

During C/SiC drilling, the machining zone is dominated by vertical axial load, while radial force components can be neglected, satisfying plane-strain conditions. According to Griffith energy release rate criterion, interlaminar crack propagation initiates once Mode I energy release rate exceeds the material intrinsic critical value *G_IC_*:

(15) $$G_{I}>G_{\mathrm{IC}}$$

Combined with Equation (14):

(16) $$G_{I}=\frac{3{F_{N}}^{2}(1-v^{2})}{8\pi^{2}Eh^{3}}=\frac{3{\left(j(\sigma +\sigma_{0}){\left(\frac{f}{v}\right)}^{2}\sin\gamma \tan\gamma \right)}^{2}(1-v^{2})}{8\pi^{2}Eh^{3}}>G_{\mathrm{IC}}$$

As interpreted from the physical meaning of Equation (16), raising the magnitude of external pre-compressive stress *σ*_0_ counteracts cutting tensile stress, lowers the net equivalent *σ* + *σ*_0_, and reduces *G_I_* to a value below *G_IC_*, thereby effectively restraining interlaminar crack nucleation and hole-exit delamination. The revised delamination analytical model identifies three feasible approaches to mitigate exit damage: optimizing processing parameters (feed rate *f* and spindle speed *v*), reducing axial drilling thrust *F_N_* by increasing pre-compressive stress *σ*_0_, and improving the material inherent critical energy release rate *G_IC_*. The complete theoretical derivation with explicit *σ*_0_ term can quantitatively predict the degree to which tuning pre-compression reduces *G_I_*, narrows the gap between *G_I_* and *G_IC_*, and correspondingly decreases the measured delamination factor. Guided by this unified mechanical logic, the adjustable pre-compression loading method offers a quantifiable and effective solution for suppressing hole-exit delamination.

Fracture mechanics experiments prove that for a fixed material under definite boundary constraints, *G_IC_* is a constant critical threshold; crack initiation and expansion will occur once *G_I_* surpasses *G_IC_*. The critical Mode I strain energy release rate *G_IC_* has different mathematical expressions under plane-strain and plane-stress states:

(17) $$G_{\mathrm{IC}}=\left\{\begin{array}{c} \frac{1-v^{2}}{E}\pi ap^{2}=\frac{1-v^{2}}{E}{K_{\mathrm{IC}}}^{2},\\ \left(in\;Plane\;Strain\;Condition\right)\\ \frac{1}{E}\pi ap^{2}=\frac{1}{E}{K_{\mathrm{IC}}}^{2},\\ \left(in\;Plane\;Stress\;Condition\right) \end{array}\right.$$

In this work, the hole-exit region bears vertical z-direction axial drilling force superimposed on uniform in-plane pre-compressive stress *σ*_0_. The combined load distributes evenly along the *z*-axis and acts uniformly on all cross-sections perpendicular to the drilling direction, conforming to the plane-strain assumption adopted above. Therefore, the critical energy release rate is simplified as:

(18) $$G_{\mathrm{IC}}=\frac{1-v^{2}}{E}\pi ap^{2}=\frac{1-v^{2}}{E}{K_{\mathrm{IC}}}^{2}$$

At this stage, the quantitative linkage among critical fracture threshold *G_IC_*, material fracture toughness *K_IC_*, and adjustable external pre-compressive stress *σ*_0_ is fully established. Combining Equation (14) and Equation (18), it is concluded that increasing the magnitude of applied *σ*_0_ significantly reduces the crack driving energy *G_I_* and widens the safety margin between *G_I_* and *G_IC_*. This theoretically verifies that applying mechanical pre-compression to generate crack-tip closure stress field is an effective technical route to improve the equivalent fracture resistance of C/SiC composites and suppress drilling-induced delamination.

### 2.3. Discussion on Model Simplification and Limitation

This theoretical model mainly focuses on Mode I interlaminar cracks responsible for hole-exit delamination. In actual drilling processes, mixed Mode I/II fracture may simultaneously take place inside C/SiC composites due to complex coupled stress fields induced by cutting edges. Nevertheless, under the constant spindle speed and feed rate adopted in all comparative tests, axial thrust force serves as the dominant load triggering hole-edge separation. The shear stress that drives Mode II crack growth is relatively weak at the exit surface, and its contribution to the final delamination factor *F*_d_ can be neglected for quantitative comparison in this research. Future work will establish a coupled I-II fracture model to achieve more accurate prediction of crack evolution.

## 3. Experimental Equipment, Instruments and Materials

### 3.1. Experimental Materials

The workpiece material adopted in this work is a 2.5D carbon fiber-reinforced silicon carbide (C/SiC) ceramic matrix composite with a carbon fiber volume fraction of 35 vol%. The interlock 2.5D woven preform is fabricated using continuous carbon fiber supplied by Toray Industries, Inc., Tokyo, Japan. A ~0.3 μm-thick BN interphase is uniformly coated on fiber surfaces via CVI to optimize interfacial bonding and prevent brittle interlayer cracking. The SiC matrix is densified by repeated PIP cycles, where the polycarbosilane precursor is sourced from National University of Defense Technology, Changsha, China. Each impregnation and pyrolysis process is conducted at 1100–1200 °C under argon protection to fill internal pores of the preform.

The final 2.5D C/SiC composite possesses a strictly balanced phase composition to ensure volumetric conservation. The calibrated volume fractions are 35 vol% carbon fiber, 6–8 vol% BN interphase, and 54–59 vol% SiC matrix. The total volume fraction of all constituent phases is 100 vol%, eliminating the unreasonable volumetric superposition issue. All residual pores and trace residual Si are included within the matrix volume range in the statistical composition. All specimens are cut from a single bulk blank by wire electrical discharge machining to ensure consistent material properties, with uniform dimensions of 100 mm × 20 mm × 7 mm.

**Figure 7:**
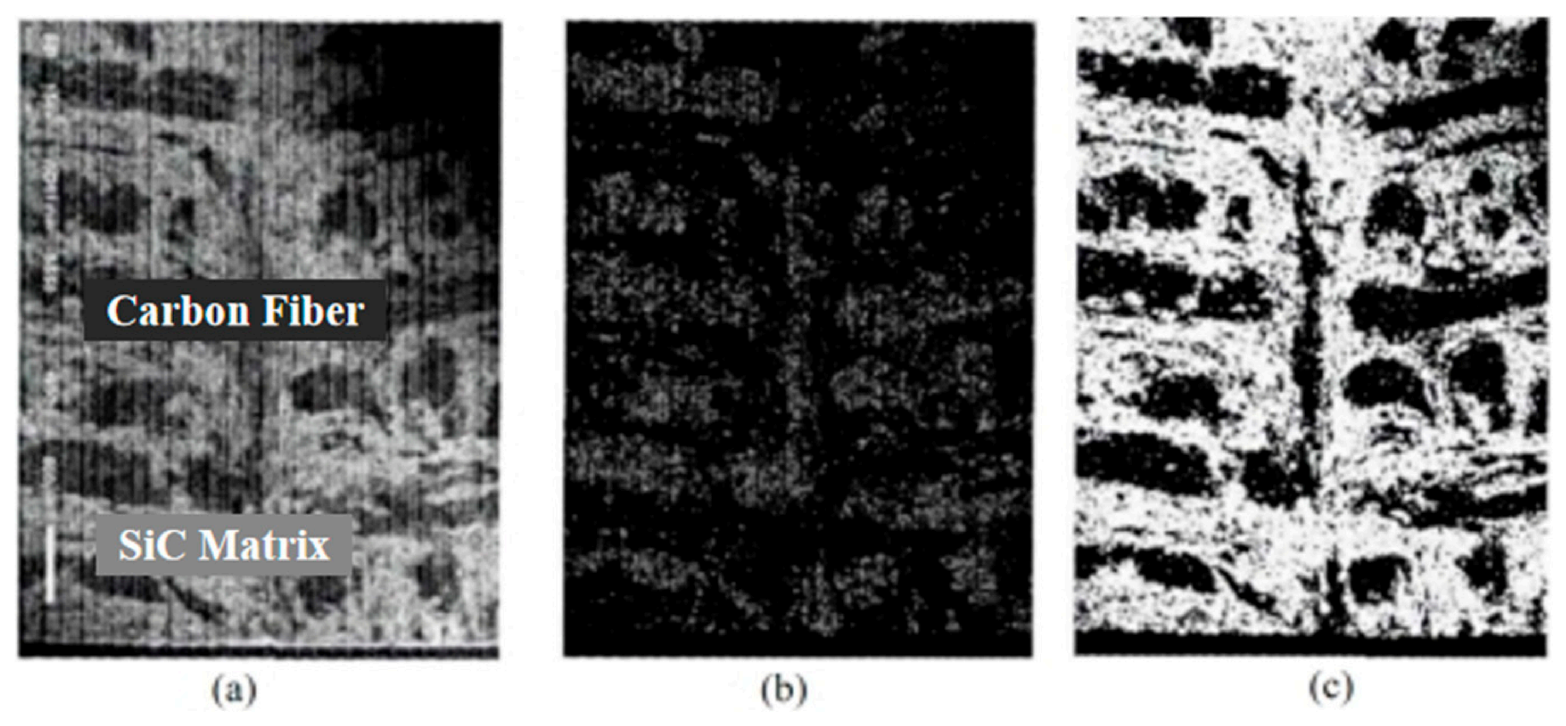
SEM morphology and EDX analysis: (**a**) SEM of cross, (**b**) distribution of C element, (**c**) distribution of Si element.

Figure 7 presents the cross-sectional SEM micrographs and corresponding EDX elemental mapping results of the composite. Specifically, Figure 7a shows the cross-sectional morphology of the as-fabricated workpiece, Figure 7b illustrates the distribution of carbon (C) element, and Figure 7c displays the distribution of silicon (Si) element. The layered stacking architecture of carbon fiber plies can be clearly observed in Figure 7a, which contributes to the improved fracture toughness of the composite. A vertical penetrating boundary line located at the central region of Figure 7a corresponds to the needle-punched structure deliberately introduced during material fabrication, aiming to strengthen the interlayer bonding strength between fiber layers. In the EDX mapping results shown in Figure 7b,c, red zones denote regions enriched in C element, while yellow zones represent areas dominated by Si element.

**Figure 8:**
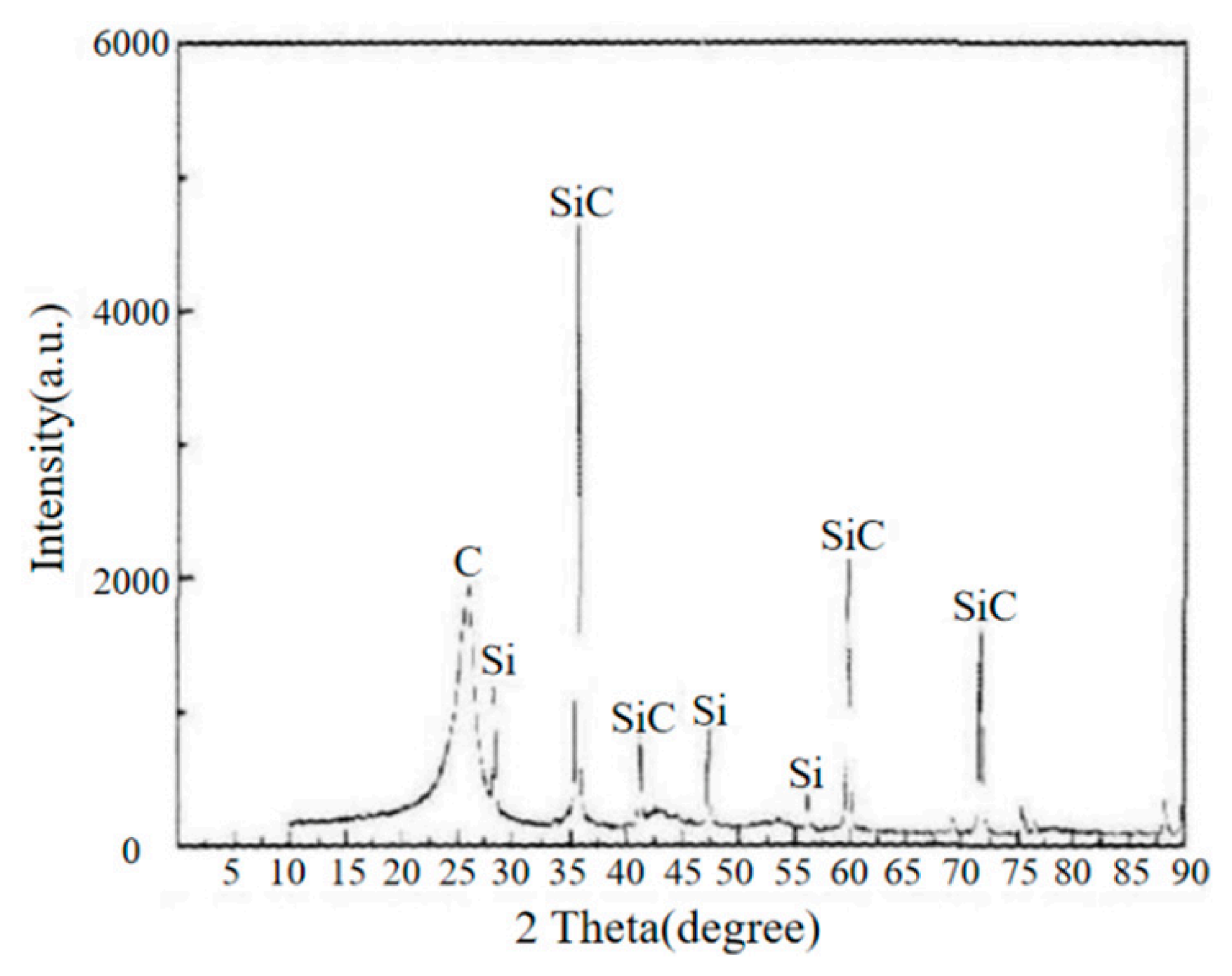
XRD analysis of C/SiC composite.

Figure 8 provides the XRD spectrum of the composite material. The diffraction results confirm that the composite is mainly composed of SiC and C phases, accompanied by a minor amount of residual Si. Comparative analysis of Figure 7a–c reveals that the black domains in the cross-sectional micrograph coincide with the distribution of C phase, whereas the gray regions match the distribution of Si-containing phases. This demonstrates that the gray matrix regions in Figure 7a consist of abundant SiC matrix and a small quantity of residual Si, and the black domains correspond to carbon fiber reinforcements. The key mechanical properties of the 2.5D C/SiC composite are summarized in Table 2 [11].

### 3.2. Experimental Tools

The silicon carbide (SiC) matrix of C/SiC ceramic matrix composites possesses ultrahigh hardness (approximately 22 GPa). Cutting tools with low hardness are unsuitable for machining C/SiC materials. Accordingly, cutting tools with extremely high hardness are mandatory for all drilling tests conducted in this work.

**Figure 9:**
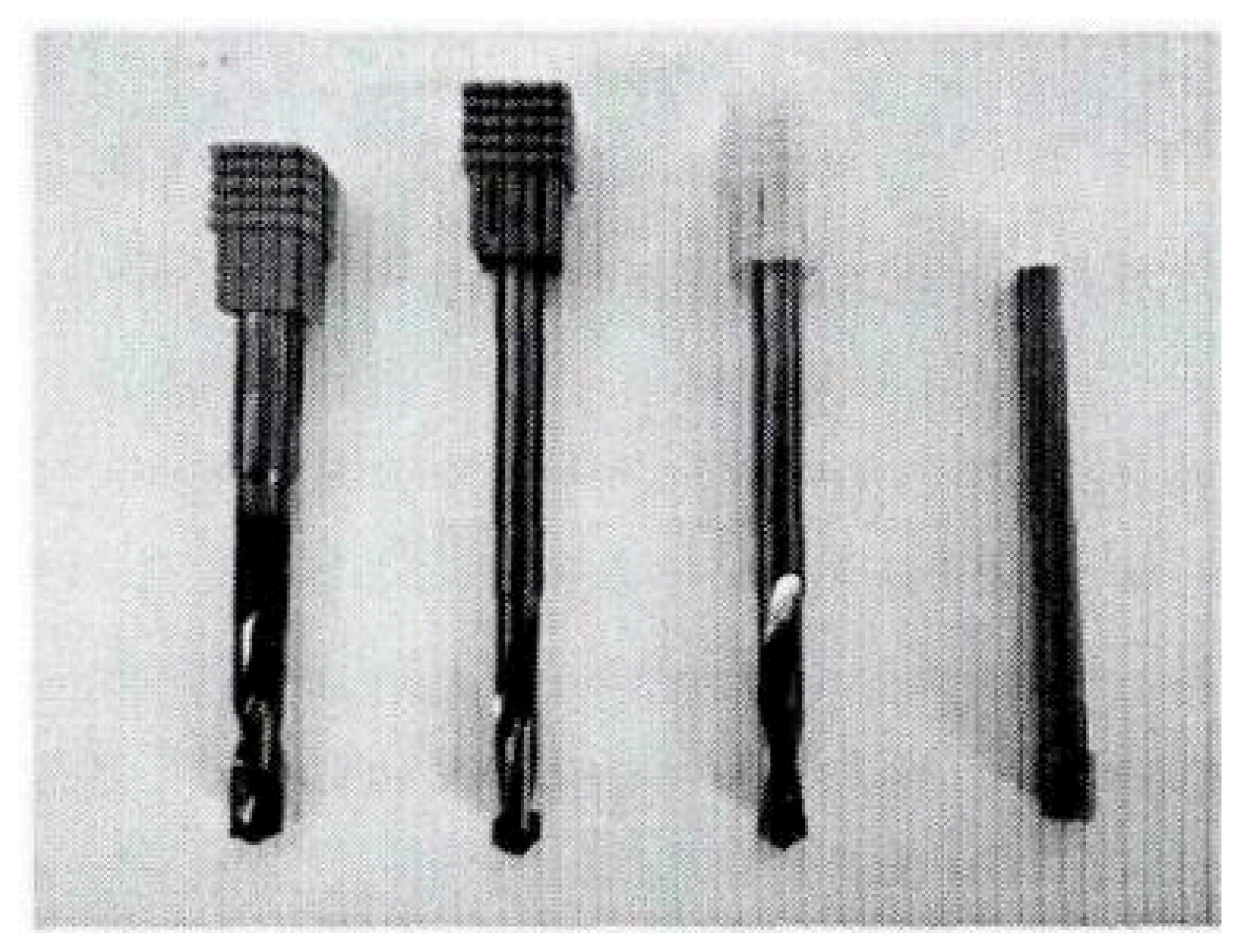
Actual drawing of experimental cutting tool.

After technical consultations with domestic cutting tool manufacturers, including Zhuzhou Cemented Carbide Cutting Tools Co., Ltd., Zhuzhou, China; Chengdu Chengliang Tools Group Co., Ltd., Chengdu, China and Xiamen Golden Egret Special Alloy Co., Ltd., Xiamen, China, it was confirmed that no commercial dedicated drill bits are available on the domestic market for drilling SiC ceramic matrix composites. Referring to the tool configurations reported in published literature [11,12,41], customized drill bits exclusively for C/SiC machining were commissioned from Xiamen Golden Egret Special Alloy Co., Ltd., as illustrated in Figure 9.

To satisfy the fundamental machining requirement that tool hardness must exceed workpiece hardness, four types of tool materials were selected for the customized drills: AlTiN coating, cubic boron nitride (CBN), polycrystalline diamond (PCD), and brazed diamond abrasives, with corresponding hardness values of 32–36 GPa, 45–50 GPa, 70 GPa and 70 GPa, respectively. All customized drill bits adopt a unified outer diameter of 6 mm to facilitate comparative analysis. As listed in Table 3, small rake angles and clearance angles were adopted for all drill geometries to mitigate edge chipping and abrasive wear.

The substrate of the AlTiN-coated drill is Cr_12_MoV alloy, on which a black AlTiN coating is deposited via physical vapor deposition (PVD). Owing to its wide application in machining high-hardness materials, this coated tool was selected for exploratory machining of ceramic matrix composites. For the brazed CBN drill, the fabrication process consists of cutting bulk CBN blanks into preforms, grinding the blanks to the target cutting edge geometry, and finally brazing the shaped CBN inserts onto a Cr_12_MoV alloy substrate. The manufacturing procedure for brazed PCD drills is fundamentally identical to that of CBN drills. The brazed diamond abrasive drill relies on high-temperature brazing technology to generate chemical metallurgical bonding among diamond grits, brazing filler metal and tool substrate. This process firmly anchors diamond abrasives around the tool periphery, with roughly half of each diamond grit exposed. By comparison, the brazed diamond abrasive drill requires neither coating deposition nor blank cutting and edge grinding procedures, delivering a relatively simplified fabrication process.

### 3.3. Experimental Equipment and Instruments

**Figure 10:**
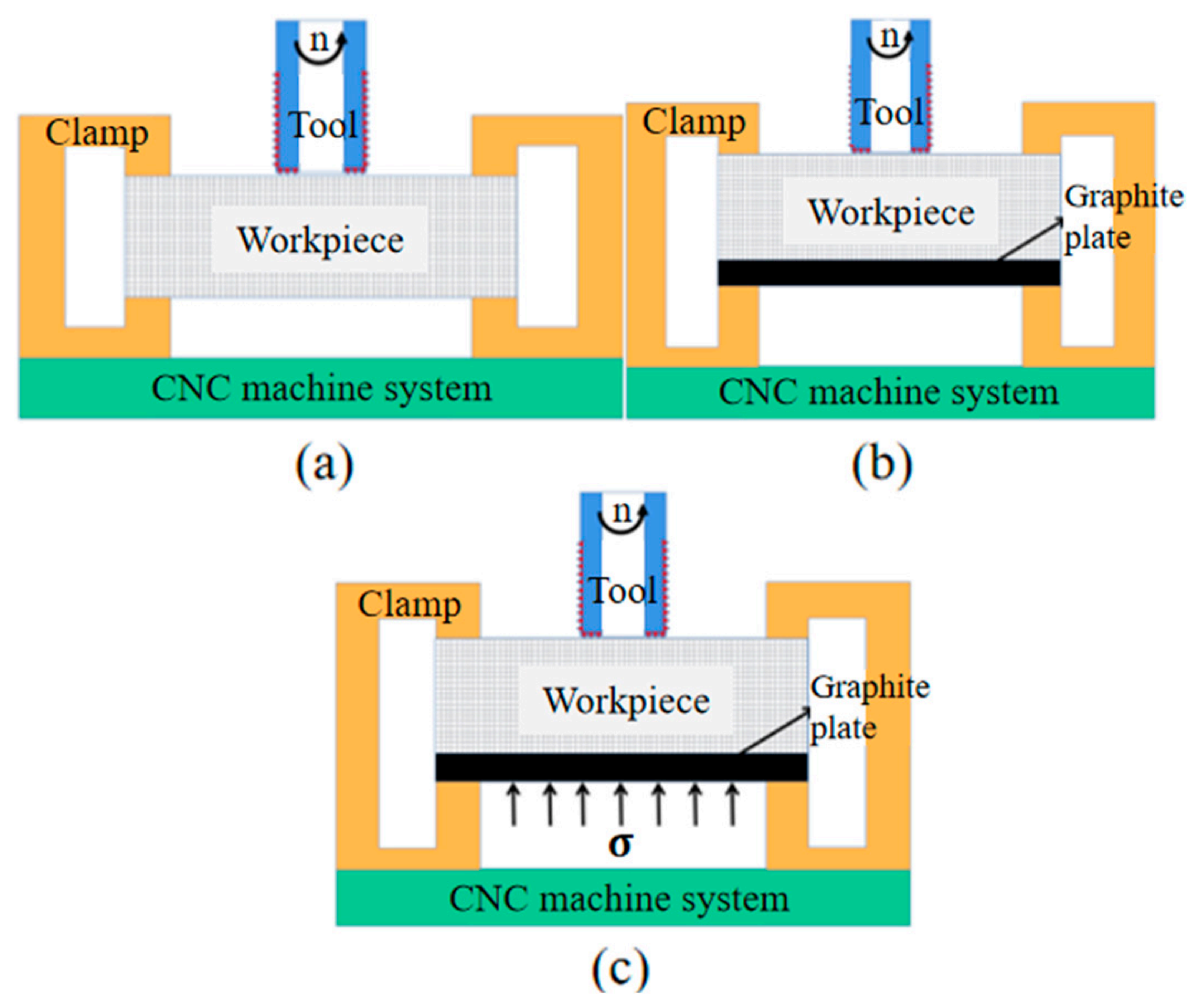
Three drilling types: (**a**) with nothing (**b**) with graphite plate (**c**) with graphite plate and torque.

Comparative drilling experiments were designed in this section to verify the theoretical hypothesis that mechanical pre-compression improves the fracture toughness of C/SiC specimens and suppresses hole-exit delamination. Two experimental configurations are illustrated in Figure 10. Experiment (a) serves as the control group, where C/SiC specimens are drilled at a spindle speed of 2500 r/min and a feed rate of 10 mm/min without any auxiliary support. In configuration (b), a graphite plate is placed under the workpiece, and adjustable compressive stress is loaded onto the specimen via a digital torque wrench, with identical drilling parameters (2500 r/min spindle speed, 10 mm/min feed rate) adopted for all machining tests.

**Figure 11:**
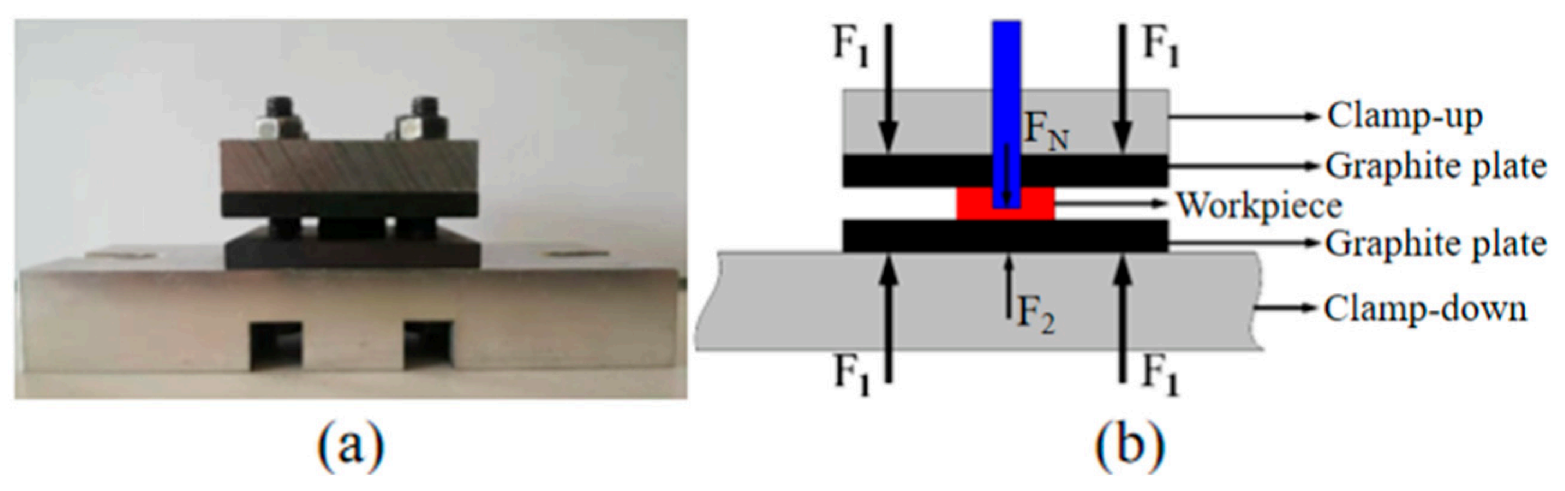
Custom clamping fixture: (**a**) physical photo; (**b**) internal force schematic.

A custom fixture capable of applying uniform controllable compressive stress to specimens was independently developed to meet the experimental requirements, as presented in Figure 11. The fixture assembly consists of an upper clamp, a lower clamp, graphite plate, bolt-nut sets and a digital torque wrench. The upper and lower clamps are used to clamp the workpiece and threadedly connect the whole fixture to the triaxial dynamometer. The graphite plate distributes homogeneous compressive stress across the specimen surface to satisfy plane-strain conditions. Bolts, nuts and the torque wrench jointly provide tunable pre-compressive stress according to different test schemes.

**Figure 12:**
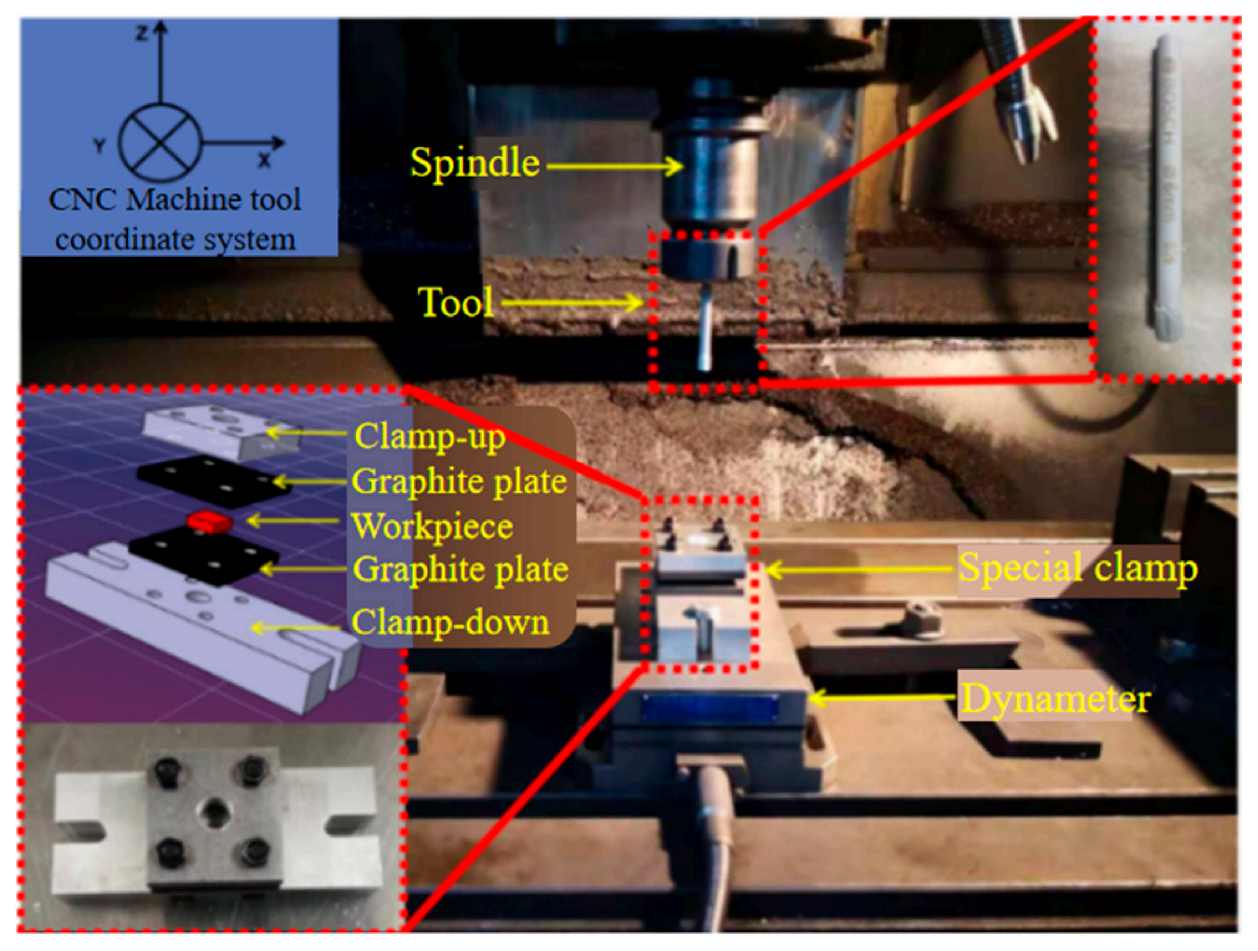
Overall layout of whole drilling experimental platform.

As shown in Figure 12, all drilling experiments were conducted on a CNC vertical milling machine (SMTCL, Shenyang, China) with a maximum spindle speed of 10,000 r/min. Brazed diamond abrasive core drills were utilized under dry drilling conditions. A Kistler 9257B triaxial piezoelectric dynamometer (Kistler Instruments AG, Winterthur, Switzerland) was mounted under the fixture to capture real-time axial cutting force signals, while a Yariki-RSX125 digital torque wrench (Yariki Precision Machinery Co., Ltd., Taichung, Taiwan, China) controlled the clamping torque. After drilling, a Keyence VHX-600E ultra-depth-of-field 3D microscope (Keyence Corporation, Osaka, Japan) was employed to characterize hole-exit morphology and quantify the delamination factor *F*_d_.

**Figure 13:**
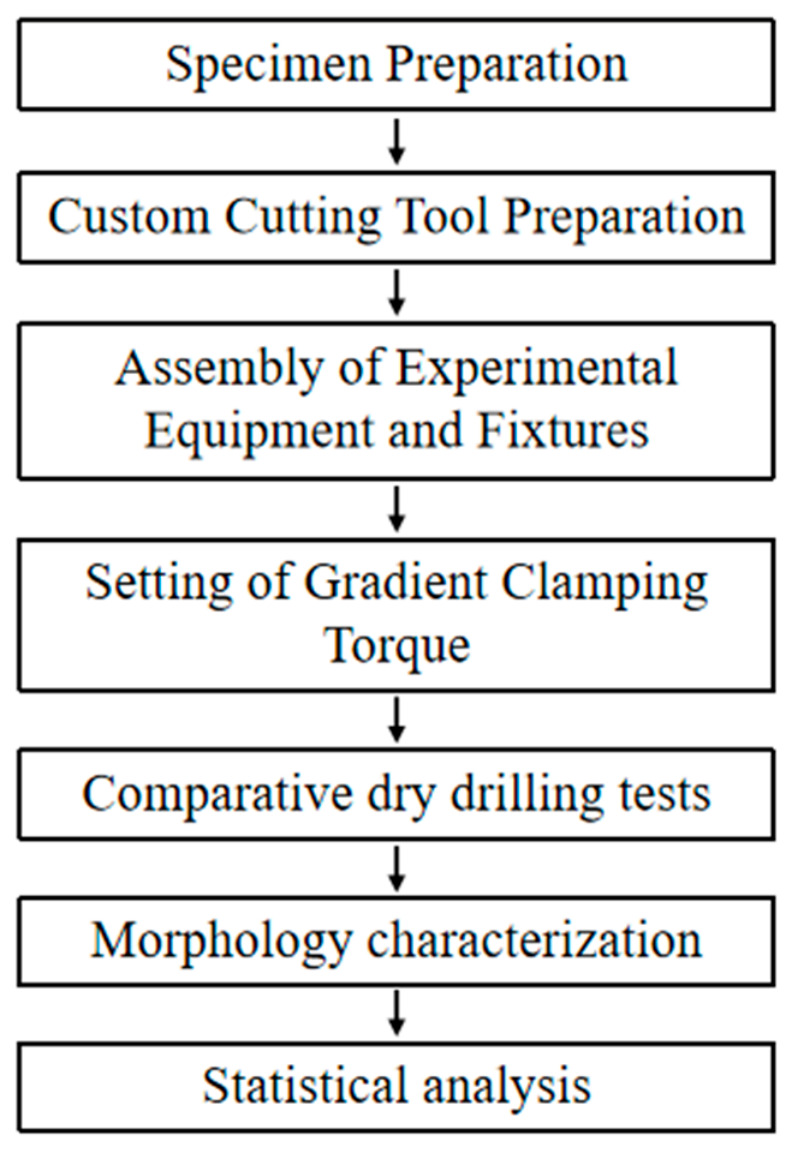
Flow chart of compressive stress-assisted drilling experiment.

To fully clarify the complete experimental workflow as suggested by the reviewer, a dedicated flowchart is supplemented as Figure 13. It is worth noting that all compressive stress values adopted in this study are purely analytical solutions derived from static mechanical formulas combined with experimental calibration, rather than finite element simulation results or direct sensor measurements. Accordingly, mesh discretization and model training are not involved in this work.

## 4. Results and Discussion

### 4.1. Evolution of Hole-Exit Morphology and Quantitative Analysis of Delamination Factor

Standardized definitions of four core terms are listed in Table 4 to maintain consistent terminology across the full text. All experiments adopted identical spindle speed of 2500 rpm and feed rate of 10 mm·min^−1^, while clamping torque was set sequentially to 0 cNm, 10 cNm, 20 cNm, 30 cNm, 40 cNm and 50 cNm. The corresponding uniform in-plane compressive stress on specimens was analytically calculated as 0 MPa, 0.46 MPa, 1.92 MPa, 3.38 MPa, 4.84 MPa, 6.30 MPa and 7.76 MPa, as listed in Table 5. The torque-to-compressive-stress conversion routine is built upon static equilibrium of the clamping fixture and elastic contact mechanics. The full set of governing equations for this conversion is fully derived and provided in Section 2 of this manuscript for complete reproducibility. Briefly, the calculation workflow proceeds as follows:

(1) Convert fixture tightening torque into bolt axial preload via the classic bolt torsion–tension correlation formula, which is explicitly given in Section 2.
(2) Distribute the total bolt preload evenly over the defined contact area between graphite plate and C/SiC workpiece to obtain initial nominal contact stress.
(3) Correct contact stress using a preload transfer coefficient calibrated through static compression pre-tests to account for minor interface friction and uneven contact.

This multi-step analytical procedure integrates fixture geometric dimensions, graphite-workpiece contact area and calibrated transfer coefficients, so all readers can replicate the 0–7.76 MPa gradient compressive stress calculation independently.

**Figure 14:**
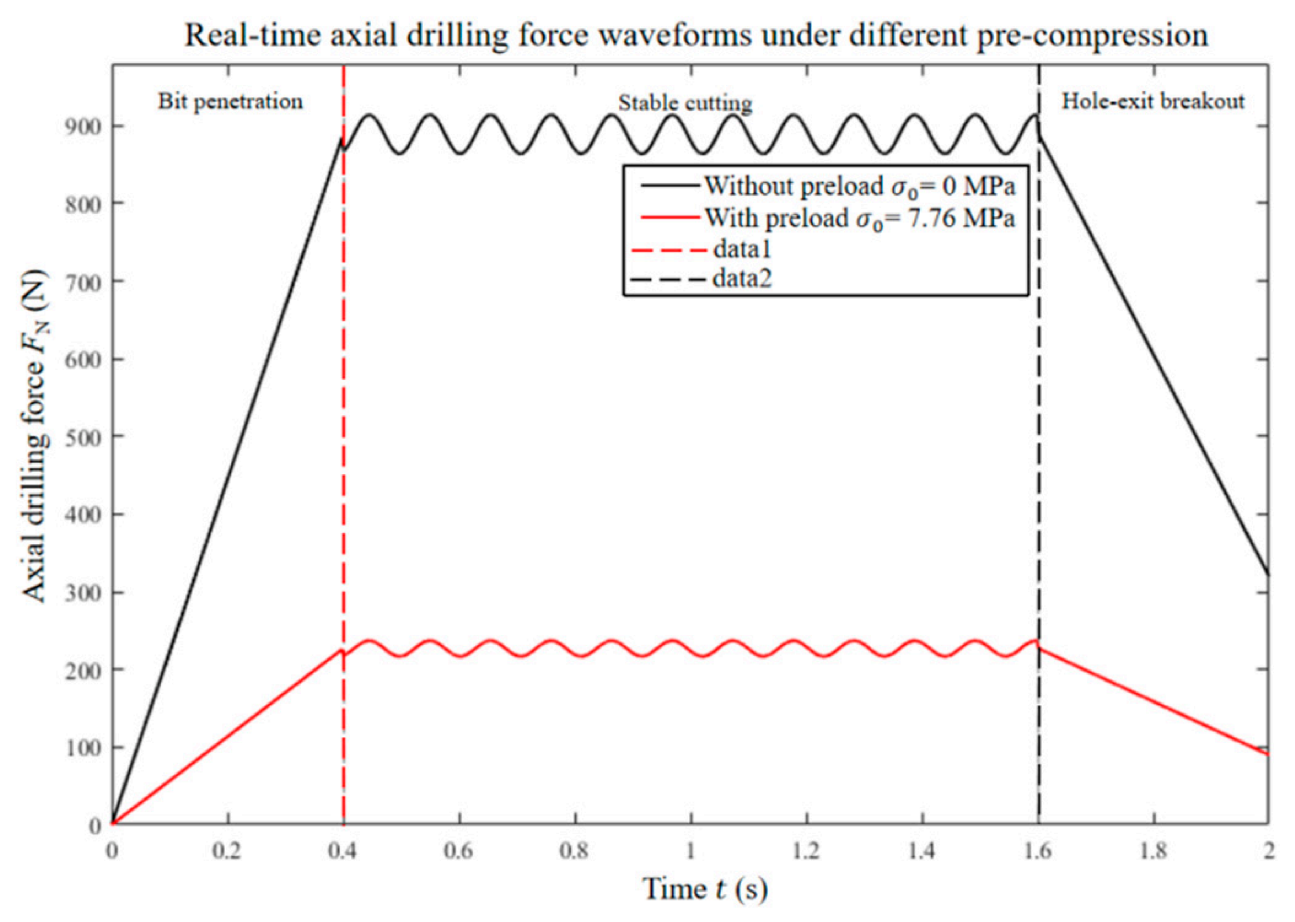
Real-time axial drilling force waveforms under two typical pre-compressive stress levels. Black solid line: specimen without adjustable preload (*σ*_0_ = 0 MPa); red solid line: specimen under the maximum pre-compressive stress (*σ*_0_ = 7.76 MPa). Vertical dashed lines divide the whole drilling process into three stages: bit penetration, stable cutting, and hole exit breakout.

Full-cycle axial drilling force signals throughout the entire machining process were continuously captured by a Kistler 9257B triaxial piezoelectric dynamometer under all experimental conditions, and one group of representative real-time axial force waveforms (Figure 14) to provide intuitive dynamic experimental evidence for the damage inhibition mechanism proposed in this work.

As illustrated in Figure 14, all test curves share identical three-stage dynamic cutting features: the axial force rises steadily during bit penetration, maintains a stable plateau in the full-diameter cutting phase, and drops sharply at the hole exit where interlaminar cracks originate. Two representative curves of the baseline non-preloaded specimen and the 7.76 MPa pre-compressed specimen are selected for comparison. The non-preloaded case presents prominent force peaks and abrupt load drop at the outlet, while pre-compressed specimens yield lower peak axial force and milder force fluctuation during the hole-exit stage. These time-domain dynamic force characteristics prove that pre-compressive stress alleviates instantaneous cutting impact and edge stress concentration, strongly supporting the crack-tip closure and delamination inhibition mechanism.

As listed in Table 6, the axial drilling force decreases monotonically with the rise of pre-compressive stress. The gradual reduction of axial cutting load under higher pre-compression directly verifies the proposed crack-tip closure damage suppression mechanism.

Multiple layers of measures guarantee the reliability of all measured data in this work:

(1) Each experiment condition was repeated three times to eliminate random measurement error. All morphological observations and dimensional measurements were performed in triplicate, with measurement uncertainties quantified by standard deviation values calculated from repeated replicates.
(2) All specimens were cut from one identical bulk C/SiC blank by wire EDM to eliminate material property discrepancy between samples.
(3) Machining parameters, clamping position and dynamometer calibration were fixed across all groups to avoid external disturbance.
(4) All averaged delamination factor and crack length differences between adjacent test groups are larger than their corresponding standard deviations, confirming the descending trends are true physical trends induced by pre-compression rather than random measurement noise.
(5) The zero-torque graphite backing test (Experiment 2) produces a 9.04% delamination reduction rate, which falls within the 12–15% range reported by Capello et al. [31] and 18% maximum reduction from Xing et al. [11]. The consistency between our baseline experimental data and published literature further cross-validates the accuracy of the measurement system and the credibility of the full dataset.

**Figure 15:**
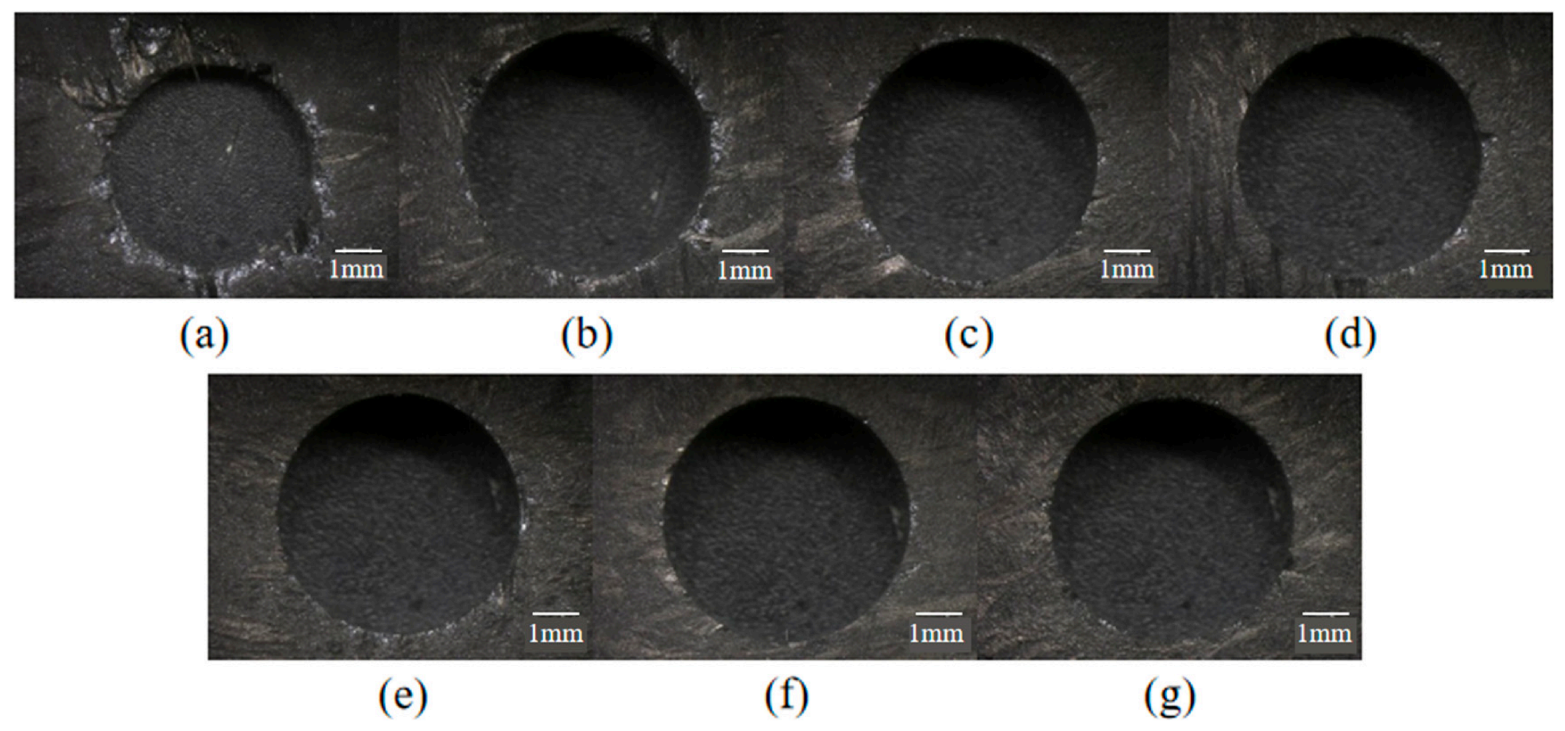
Surface topographies of hole-exit at different experiment conditions: (**a**) without graphite, (**b**) with graphite plate, (**c**) with graphite plate and 10 cNm torque, (**d**) with graphite plate and 20 cNm torque. (**e**) with graphite plate and 30 cNm torque, (**f**) with graphite plate and 40 cNm torque, (**g**) with graphite plate and 50 cNm torque.

**Figure 16:**
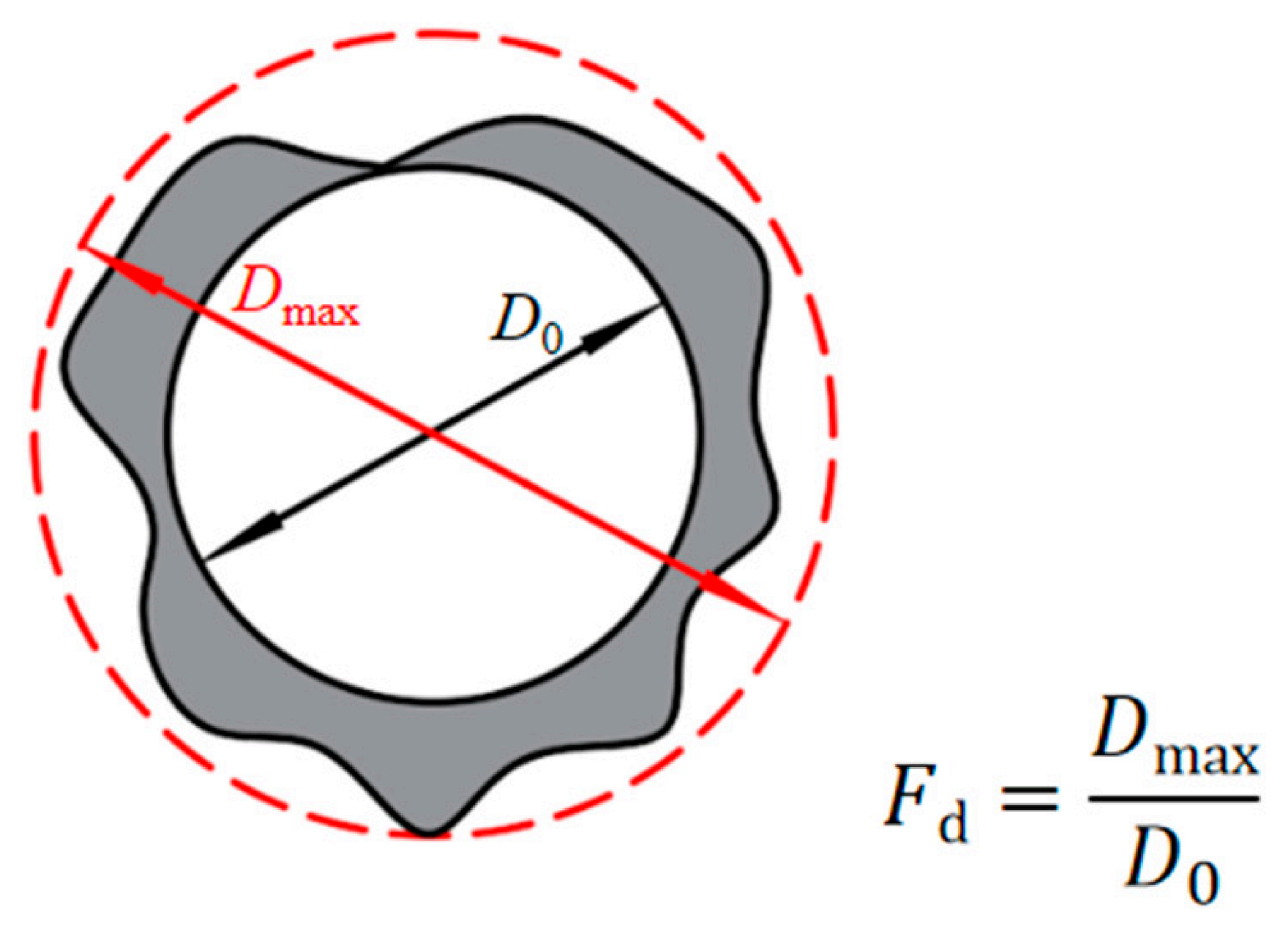
Schematic diagram for delamination factor definition.

Figure 15 displays hole-exit surface topographies under seven experiment conditions, where delamination defects shrink gradually with rising preload. The delamination factor *F*_d_ is adopted to quantify damage severity. As defined in Figure 16, *F*_d_ = *D*_max/_*D*_0_, where *D*_max_ is the diameter of the circumscribed circle covering all delamination zones, and *D*_0_ = 6 mm is the nominal drill diameter [30].

**Figure 17:**
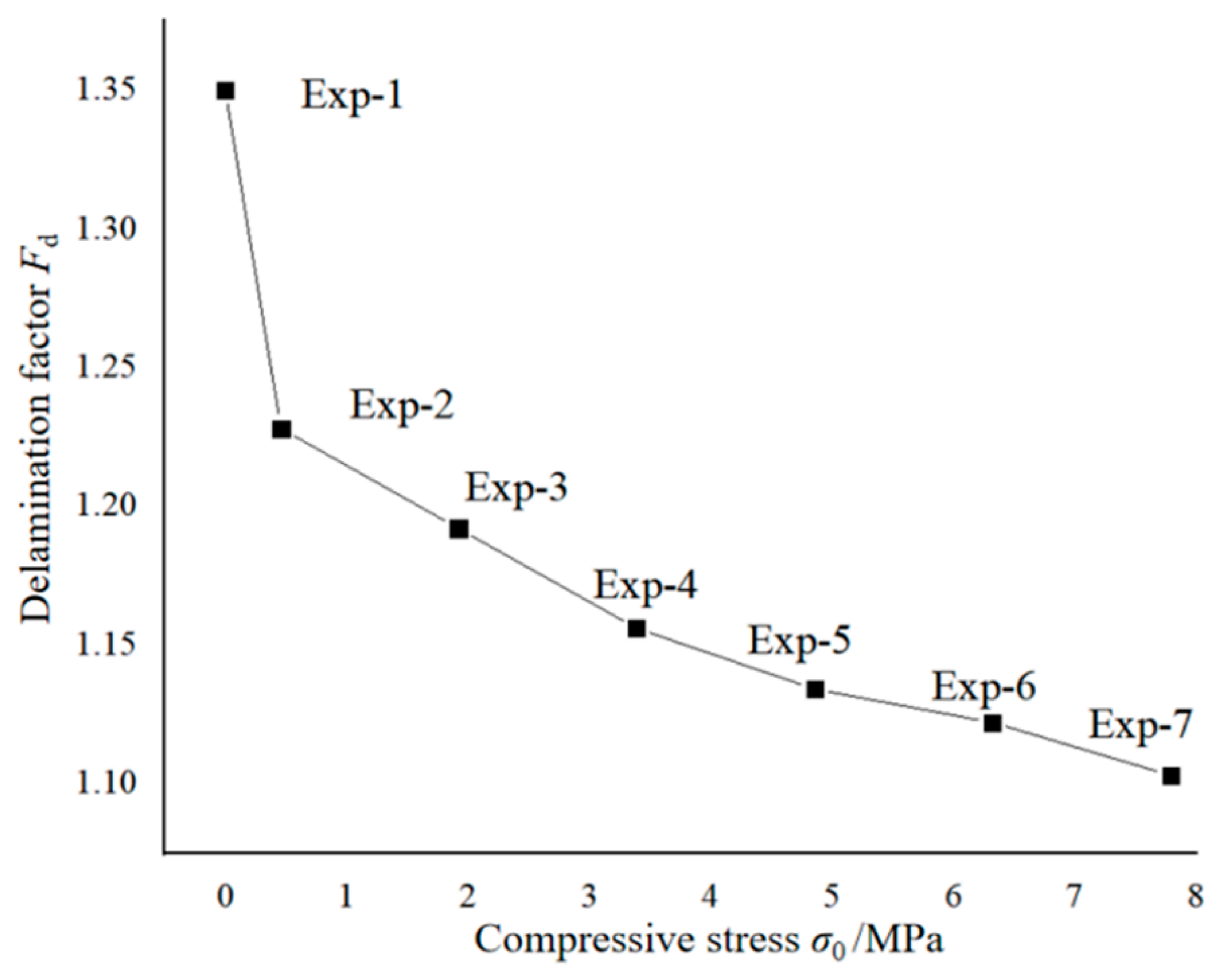
Effect of compressive stress on delamination damage (error bars represent standard deviation from three repeated measurements).

The average *F*_d_ values measured by an ultra-depth-of-field 3D microscope are summarized in Table 7 and plotted in Figure 17. An obvious negative correlation can be observed between pre-compressive stress and delamination factor: *F*_d_ declines from 1.350 (Experiment 1, *σ*_0_ = 0 MPa) to 1.103 (Experiment 7, *σ*_0_ = 7.76 MPa), achieving a maximum reduction ratio of 18.29%. The evolution curve of Fd exhibits a two-stage feature. Delamination damage is suppressed rapidly when *σ_0_* < 4.84 MPa, while further increasing preload above 4.84 MPa only brings marginal improvement on damage inhibition.

Table 8 lists the average maximum interlaminar crack length and corresponding standard deviation from three repeated tests, and the crack length variation curve is also drawn in Figure 17. The maximum crack propagation length decreases monotonically with rising pre-compressive stress, falling from 412.6 μm (Experiment 1) to 230.4 μm (Experiment 7), with a total reduction rate of 44.16%. The crack length curve follows the identical two-stage evolution law as the delamination factor curve. The gap of average crack length between successive stress levels is larger than the matching standard deviation, which demonstrates a stable and distinguishable downward trend of crack extension length as preload increases.

### 4.2. Statistical Analysis of Axial Drilling Force and Hole-Exit Delamination Defects

**Figure 18:**
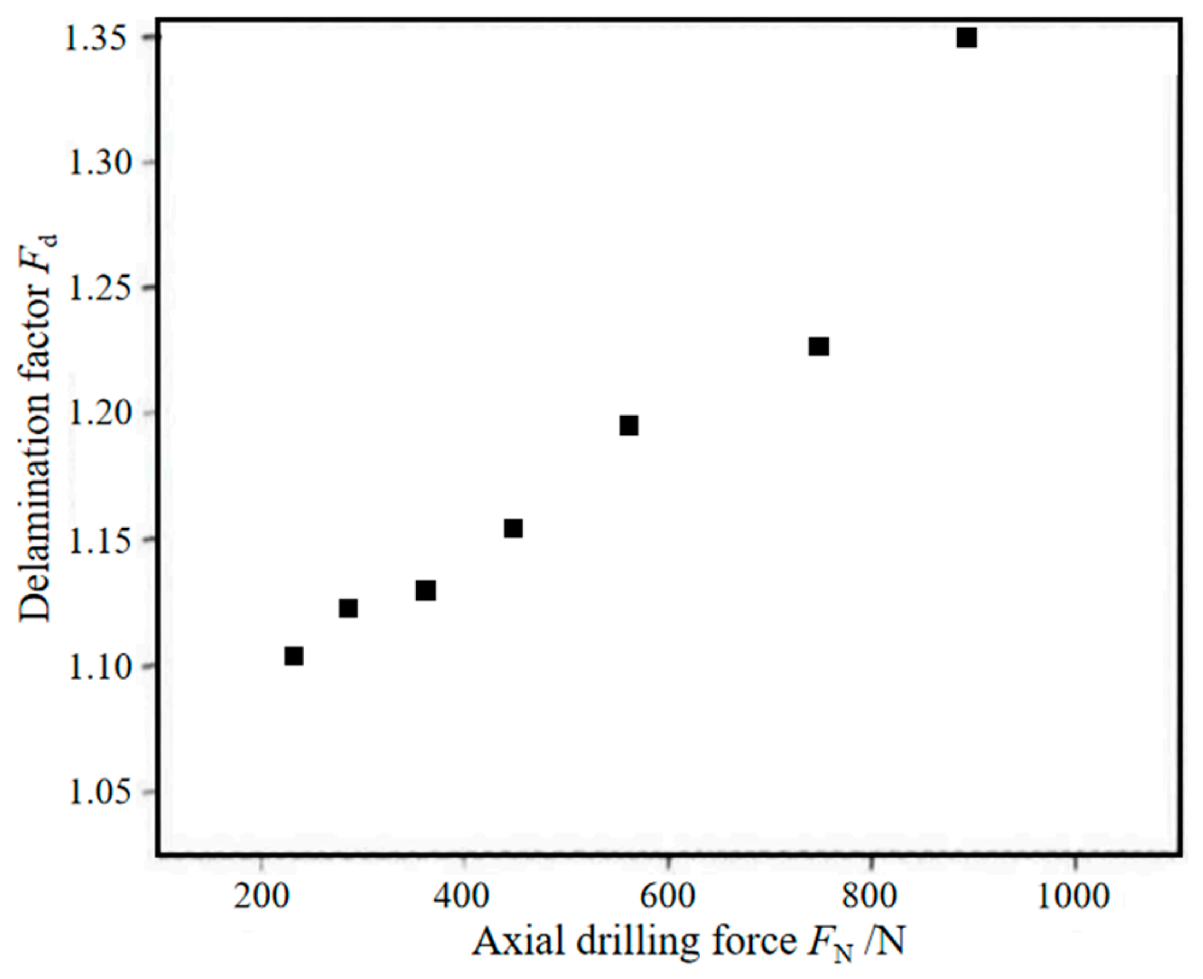
Corresponding scatter diagram of axial drilling force and hole-exit delamination factor.

From the qualitative morphological and quantitative damage analysis in Section 4.1, it is confirmed that the application of gradient pre-compressive stress can effectively reduce axial drilling force and mitigate hole-exit delamination defects in C/SiC composite drilling. To explore the inherent covariation characteristic between axial cutting load and delamination severity, paired experimental data of axial drilling force *F*_N_ and delamination factor *F*_d_ under seven pre-compressive stress levels were extracted for correlation analysis. The paired datasets and corresponding scatter distribution are summarized in Table 9 and Figure 18, respectively.

Visual observation of the scatter plot demonstrates a distinct positive linear trend, where a higher axial drilling force generally corresponds to aggravated hole-exit delamination. To quantitatively characterize this covariation relationship, descriptive statistical analysis and two-tailed Pearson correlation tests were performed, with the statistical results presented in Table 10 and Table 11.

The statistical results show a strong positive linear correlation (r = 0.963, *p* = 0.001) between axial drilling force and delamination factor across the seven tested conditions. Notably, this significant correlation only reflects a robust linear covariation trend between the two physical variables. It does not indicate statistically significant differences in delamination performance or drilling force between individual pre-compressive stress groups. In this work, one-way ANOVA or pairwise comparative statistical tests were not conducted to quantitatively verify inter-group statistical discrepancies. The gradual descending trends of *F*_d_ and crack length with increasing preload described in Section 4.1 are qualitatively validated by comparing average values and corresponding standard deviation ranges, rather than rigorous inter-group statistical significance verification.

Combining the qualitative damage evolution law in Section 4.1, dynamic force waveform characteristics, and quantitative correlation results in this section, the intrinsic mechanism of pre-compressive stress assisted delamination suppression is fully clarified. The externally applied in-plane compressive stress constructs a stable crack-tip closure stress field during drilling, which offsets part of the cutting-induced tensile stress and reduces the effective axial drilling force acting on the interlaminar interface. The decreased axial cutting load restrains the initiation and propagation of Mode I interlaminar cracks at the hole exit, thereby steadily reducing the delamination factor and maximum crack length. The consistent experimental trends and statistical correlation results mutually verify the feasibility and reliability of the pre-compression-assisted drilling optimization strategy for C/SiC composites.

### 4.3. Mechanism Comparison Between Other Unloaded Graphite Plate Drilling and Proposed Pre-Compression Drilling

To verify the effectiveness and technological advancement of the proposed pre-compression-assisted drilling method, this section systematically compares its mechanical mechanisms and machining performance with conventional zero-preload graphite-assisted drilling reported in existing studies. Multi-dimensional comparisons covering stress generation, delamination suppression principles, quantitative adjustability, machining performance and engineering applicability are summarized in Table 12, which quantitatively clarifies the inherent differences and prominent advantages of the proposed method over traditional similar technologies.

The baseline experimental results of zero-preload graphite drilling in this work are highly consistent with the conclusions of existing composite and ceramic matrix composite (CMC) drilling studies, which validates the reliability and repeatability of the experimental system. Previous studies have confirmed that passive graphite backing can effectively suppress drilling-induced delamination, with a reported damage reduction rate of 12–18% [11,31]. In this work, the zero-preload graphite group achieves a 9.04% delamination reduction rate, which falls within a reasonable error range of published data and conforms to the universal passive support suppression law. The consistent qualitative trends and quantitative results demonstrate the comparability and credibility of the experimental data in this study.

Mechanistically, the passive suppression effect of graphite plates observed in this work further validates two classical delamination suppression theories widely recognized in composite drilling research. Consistent with the critical thrust force model proposed by Hocheng and Tsao [34,35], the rigid graphite support shares axial drilling extrusion load and increases the critical thrust for interlaminar crack initiation. Additionally, the contact constraint between the graphite plate and the C/SiC workpiece restricts bottom ply bending and homogenizes edge tensile stress distribution, which matches the deformation control mechanism proposed by Xing et al. [11]. Nevertheless, the passive graphite support adopted in conventional studies only delays initial crack nucleation through fixed contact reaction force, suffering from non-adjustable and limited suppression performance.

**Figure 19:**
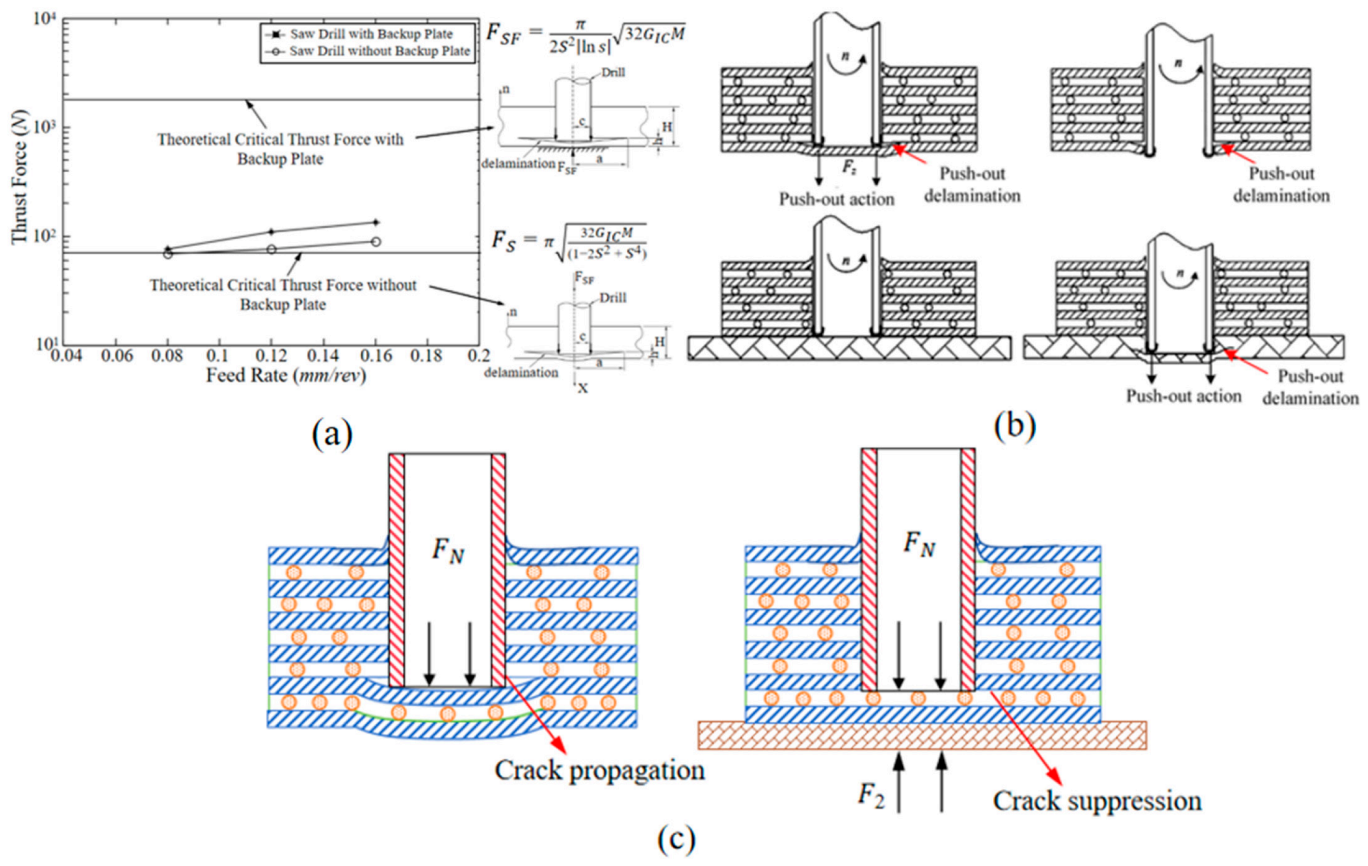
The role of the graphite plate from different angle: (**a**) critical thrust force (**b**) push-out action (**c**) compressive stress.

Different from the passive fixed constraint mode of traditional graphite-assisted drilling, this work proposes an active torque-preloaded compression strategy with innovative mechanical mechanisms. Existing studies only focus on the passive functions of graphite plates, including critical thrust improvement and push-out deformation limitation (Figure 19a,b). By contrast, the proposed method realizes uniform and adjustable in-plane compressive stress distribution around the hole exit (Figure 19c). The gradient preload constructs a stable crack-tip closure stress field, which inhibits the entire propagation process of Mode I interlaminar cracks, rather than merely delaying crack initiation as realized by conventional passive methods.

Quantitative comparison with existing similar studies further verifies the performance superiority of the proposed method. Conventional passive graphite drilling can only achieve fixed limited delamination suppression. In comparison, the pre-compression-assisted strategy realizes continuous gradient regulation of compressive stress, yielding a maximum delamination factor reduction of 18.29%. This value nearly doubles the suppression effect of passive graphite support in the baseline test and surpasses the fixed performance ceiling of traditional graphite-assisted drilling. Moreover, the active preloading mode eliminates the strict flat fitting requirement for workpiece bottom surfaces, enabling reliable machining of thin, curved and miniature C/SiC components that are difficult to process via conventional passive support methods.

In summary, the pre-compression-assisted delamination suppression mechanism proposed in this study complements and extends the classical theories of graphite-assisted composite drilling, without conflicting with existing research conclusions. Different from the fixed passive constraint of traditional methods, the tunable pre-compression strategy achieves quantitative, adjustable and high-efficiency delamination suppression, effectively breaking through the performance limitations and application constraints of conventional graphite-assisted drilling technologies for C/SiC composites.

## 5. Conclusions

This work primarily explores the potential delamination suppression effect induced by backside compressive preload during the drilling of C/SiC composites and attempts to clarify the correlation characteristics between axial drilling load and hole-exit interlaminar damage. Through a series of single-variable preloading drilling tests and Pearson statistical correlation analysis, the preliminary findings of this work are summarized as follows, with certain conditional applicability and room for further improvement:

(1) Backside uniform compressive preload is capable of generating a crack-tip closure stress field near the hole exit, which contributes to a gradual reduction in steady-state axial drilling thrust applied on the interlaminar interface. Within the investigated pre-stress range of 0–7.76 MPa, the measured axial drilling force decreases from 889 N to 227 N under the present experimental conditions. The reduced axial load may weaken the driving tendency of interlaminar crack initiation and thereby assist damage suppression.
(2) The externally applied pre-compressive stress mainly modulates the effective stress state at the crack tip without substantially changing the inherent Mode I interlaminar fracture toughness *G_IC_* of C/SiC composites. The introduced pre-stress can partially offset drilling-induced tensile stress, reduce the net stress intensity for crack propagation, and alleviate the degree of hole-exit delamination. The suppression effect exhibits a staged variation tendency under gradient preload: obvious damage mitigation can be observed when the pre-compressive stress is lower than 4.84 MPa, whereas further preload increment yields limited additional improvement. Within the test range, the delamination factor decreases gradually from 1.350 to 1.103 (maximum reduction of 18.29%), and the maximum interlaminar crack length reduces from 412.6 μm to 230.4 μm, indicating a favorable suppression tendency.
(3) Statistical analysis demonstrates a strong positive linear correlation (r = 0.963, *p* = 0.001) between axial drilling force and delamination factor under the tested working conditions. This correlation suggests that the pre-compression-induced reduction of axial cutting load is likely a key physical pathway for relieving hole-exit delamination, though such a coupling relationship remains valid within the present parameter window.

Furthermore, the proposed torque-to-compressive-stress conversion model is established based on static mechanical equilibrium and experimental calibration, which avoids complex finite element discretization or intelligent algorithm training. This simplified method can provide a feasible reference for preload fixture design and parameter debugging, and may offer preliminary mechanical understanding for the low-damage machining of C/SiC components. It is worth noting that the present conclusions are summarized under limited machining parameters and fixture configurations, and extended parametric verification will be required in future work to generalize the applicability of the proposed pre-compression-assisted drilling method.

## Figures and Tables

**Table 1:** The key drilling process parameters and their corresponding symbols.

Drilling Process Parameters	Corresponding Symbols
Length of the generatrix involved in cutting	*ρ*
Cone half angle	*γ*
Angle between microelements and cutting direction	*φ*
Stress act on micro area	*σ*
The number of all abrasive particles involved in grinding	*j*
Feed rate	*f*
Rotation speed	*v*
Crack strain energy release rate per unit area	*G_I_*
Radius of the crack	*a*
With the axial drilling force moves down by displacement	*X*
Unit stiffness	*M*
Strain energy stored in the crack area (circle below the tool) where deformation occurs in the workpiece	*U*
Modulus of elasticity	*E*
Poisson’s ratio	*ν*
Drilled section thickness	*h*
Critical crack strain energy release rate	*G_IC_*

**Table 2:** Physical and mechanical properties of C/SiC.

Parameter Names	Numerical Value
Density/$g\cdot {\mathrm{cm}}^{-3}$	1.9
Porosity %	5
Tensile strength/MPa	87.5
Compressive strength/MPa	460
Bending strength/MPa	260
Elasticity modulus/GPa	40
Interlaminar shear strength/MPa	20
Fracture toughness/$\mathrm{MPa}\cdot m^{1/2}$	10
SIC matrix hardness/GPa	22.2
Poisson’s ratio	0.2

**Table 3:** Experimental tools and parameters.

Drill Tool Name	Parameters
AlTiN-coated drill	Model D998-Y3N, outer diameter: 6 mm, point angle: 140°, coating hardness: 32–36 GPa
Brazed CBN drill	Custom-made, outer diameter: 6 mm, point angle: 140°, rake angle: 0°, clearance angle: 6°, hardness: 45–50 GPa
Brazed PCD drill	Custom-made, outer diameter: 6 mm, point angle: 140°, rake angle: 0°, clearance angle: 6°, hardness: approximately 70 GPa
Brazed diamond abrasive core drill	Custom-made via high-temperature brazing, inner diameter: 3 mm, outer diameter: 6 mm, average diamond grit size: 233 μm, hardness: approximately 70 GPa

**Table 4:** Standard definitions of core terminology.

Term	Standard Definition
Graphite plate	Interchangeable graphite rigid support under workpiece.
Supporting force	Fixed passive contact force only induced by drill thrust (non-adjustable).
Preload	Adjustable bolt clamping torque (active control input)
Compressive stress	Tunable in-plane stress generated by preload for crack suppression.

**Table 5:** Experimental design parameters and calculated total compressive stress values.

Experiments	Spindle Speed (rpm)	Feed Rate ($mm\cdot {min}^{-1}$)	With Graphite Plate or Not	Torque (cNm)	Total Compressive Stress (MPa)
1	2500	10	without	0	0
2	2500	10	with	0	0.46
3	2500	10	with	10	1.92
4	2500	10	with	20	3.38
5	2500	10	with	30	4.84
6	2500	10	with	40	6.30
7	2500	10	with	50	7.76

**Table 6:** Measured axial drilling force indexes under different pre-compressive stress.

Experiments Number	Compressive Stress *σ*_0_ (MPa)	Axial Drilling Force *F*_N_/N
1	0	889
2	0.46	745
3	1.92	557
4	3.38	436
5	4.84	368
6	6.30	287
7	7.76	227

**Table 7:** Experimental delamination factor data under different compressive stress levels.

Experiments Number	1	2	3	4	5	6	7
*σ*_0_	0	0.46	1.92	3.38	4.84	6.30	7.76
*F*_d_	1.350	1.228	1.192	1.156	1.134	1.122	1.103
Decreased	0	9.04%	11.70%	14.37%	16.00%	16.89%	18.29%

**Table 8:** Statistical data of average maximum interlaminar crack length under different compressive stress.

Experiments Number	1	2	3	4	5	6	7
Compressive stress *σ*_0_/MPa	0	0.46	1.92	3.38	4.84	6.30	7.76
Average maximum crack length/μm	412.6	358.3	321.5	287.2	261.9	245.7	230.4
Standard deviation/μm	18.5	15.2	13.1	10.8	9.6	8.3	7.1

**Table 9:** Paired data of axial drilling force and delamination factor.

Experiments Number	1	2	3	4	5	6	7
Axial drilling force *F*_N_/N	889	745	557	436	368	287	227
Delamination factor *F*_d_	1.350	1.228	1.192	1.156	1.134	1.122	1.103

**Table 10:** Descriptive statistics.

	Average	Standard Deviation	Number of Samples
Axial drilling force *F*_N_	501.2857	243.5848	7
Delamination factor *F*_d_	1.1836	0.0849	7

**Table 11:** Pearson correlation analysis results.

	Statistical Parameters	Axial Drilling Force	Delamination Factor
Axial drilling force *F*_N_	Pearson correlation coefficient	1	0.963
	Bilateral significance (*p*-value)		0.001
	Sum of squares and cross products	356,001.428	119.523
	Covariance	59,333.571	19.9205
	Number of samples	7	7
Delamination factor *F*_d_	Pearson correlation coefficient	0.963	1
	Bilateral significance (*p*-value)	0.001	
	Sum of squares and cross products	119.523	0.04324
	Covariance	19.9205	0.00721
	Number of samples	7	7

**Table 12:** Comparative analysis of two graphite-assisted drilling methods.

Comparison Dimension	Other Unloaded Graphite Plate Drilling	Proposed Compressive Stress-Assisted Drilling Method
Generation of compressive stress	Passive contact stress generated only by drill axial extrusion; fixed magnitude of 0.46 MPa, unable to adjust independently	Active pre-compression loaded by fixture torque; continuous adjustable stress range of 0–7.76 MPa, independent of drilling thrust
Delamination suppression mechanism	Increases critical axial thrust via reverse support reaction, only delays the initial nucleation of interlayer cracks	Forms crack-tip closure stress field, restrains the whole propagation process of Mode I cracks and raises critical strain energy release rate *G_IC_*
Quantitative adjustability of suppression effect	Fixed inhibition effect, no further optimization space	Gradient controllable delamination suppression performance, matching stress according to machining accuracy requirements
Maximum reduction rate of delamination factor *F*_d_	9.04% (only from 1.350 to 1.228)	18.29% (from 1.350 down to 1.103 at 7.76 MPa preload)
Applicable workpiece conditions	Requires complete flat fitting between workpiece bottom and graphite plate; not suitable for thin, curved and small-size specimens	Applies uniform pre-compression on both sides of the workpiece, no strict flatness requirement for the back surface, compatible with thin and miniature C/SiC parts
Core functional carrier definition	Regards the graphite plate as the key factor to suppress delamination	Clarifies that the graphite plate merely acts as a uniform force transfer medium; adjustable pre-compressive stress is the dominant control variable

## Data Availability

The original contributions presented in this study are included in the article. Further inquiries can be directed to the corresponding author.
